# Tolerance and Safety Evaluation in a Large Cohort of Healthy Infants Fed an Innovative Prebiotic Formula: A Randomized Controlled Trial

**DOI:** 10.1371/journal.pone.0028010

**Published:** 2011-11-30

**Authors:** Pasqua Piemontese, Maria L. Giannì, Christian P. Braegger, Gaetano Chirico, Christoph Grüber, Josef Riedler, Sertac Arslanoglu, Margriet van Stuijvenberg, Günther Boehm, Jürgen Jelinek, Paola Roggero, for the MIPS 1 Working Group Berlin, Frankfurt (Oder), and Friedrichsdorf, Germany, Groningen, The Netherlands, Milan and Brescia, Italy, Zurich, Switzerland, and Schwarzach, Austria

**Affiliations:** 1 Department of Maternal and Pediatric Sciences, Fondazione IRCCS ‘‘Ca'Granda’’ Ospedale Maggiore Policlinico, University of Milan, Milano, Italy; 2 Division of Paediatric Gastroenterology and Nutrition, University Children's Hospital, Zurich, Switzerland; 3 Department of Neonatology, Spedali Civili, Brescia, Italy; 4 Department of Pediatric Pneumology and Immunology, Charité-Universitäts- Medizin, Berlin, Germany; 5 Children's Hospital Schwarzach, Schwarzach/Pg, Austria; 6 Centre for Infant Nutrition, Hospital Macedonio Melloni, Milan, Italy; 7 Division of Neonatology, Beatrix Children's Hospital, Groningen, The Netherlands; 8 Danone Research Center for Specialized Nutrition, Friedrichsdorf, Germany; Aga Khan University, Pakistan

## Abstract

**Background:**

the addition of oligosaccharides to infant formula has been shown to mimic some of the beneficial effects of human milk. The aim of the study was to assess the tolerance and safety of a formula containing an innovative mixture of oligosaccharides in early infancy.

**Methodology/Principal Findings:**

this study was performed as a multi-center, randomized, double-blind, placebo-controlled trial including healthy term infants. Infants were recruited before the age of 8 weeks, either having started with formula feeding or being fully breast-fed (breastfeeding group). Formula-fed infants were randomized to feeding with a regular formula containing a mixture of neutral oligosaccharides and pectin-derived acidic oligosaccharides (prebiotic formula group) or regular formula without oligosaccharides (control formula group). Growth, tolerance and adverse events were assessed at 8, 16, 24 and 52 weeks of age. The prebiotic and control groups showed similar mean weight, length and head circumference, skin fold thicknesses, arm circumference gains and stool frequency at each study point. As far as the anthropometric parameters are concerned, the prebiotic group and the control group did not attain the values shown by the breastfeeding group at any study point. The skin fold thicknesses assessed in the breastfeeding group at 8 weeks were strikingly larger than those in formula fed infants, whereas at 52 weeks were strikingly smaller. The stool consistency in the prebiotic group was softer than in the control group at 8, 16 and 24 weeks (p<0.001) and closer to that of the breastfeeding group. There was no difference in the incidence of adverse events between the two formula groups.

**Conclusions:**

our findings demonstrate the tolerability and the long term safety of a formula containing an innovative mixture of oligosaccharides in a large cohort of healthy infants.

**Trial Registration:**

drks-neu.uniklinik-freiburg.de DRKS 00000201

## Introduction

Several of the health benefits related to breastfeeding have been ascribed to oligosaccharides that modulate the development of a typical gut microbiota [1]. Indeed, they play a major role in generating a microbiota characterized predominantly by the presence of bifidobacteria and lactobacilli [2]. On the contrary, the microbiota of formula-fed infants compromises more Bacteroides, Staphylococci, Escherichia Coli and Clostridia [3]. There is evidence that the development of bifidobacteria predominant microbiota may favour a decreased incidence of infections, allergies and gastrointestinal symptoms as seen in breast-fed infants compared with formula-fed infants [4]. On the basis of the available data, for infants who are not breastfed, it seems reasonable to supplement infant formulas with oligosaccharides in order to develop intestinal microbiota similar to that of breastfed infants.

In human milk 80–85% of the oligosaccharides are neutral and 15–20% are acidic. A specific mixture of neutral oligosaccharides of non-milk origin has been developed to mimic the neutral oligosaccharides fraction of human milk, namely, short chain galacto-oligosaccharides which derived from enzymatic conversion of lactose and long chain fructo-oligosaccharides from chicory derived inulin [5]. The human acidic-oligosaccharides composition is different from the composition of acidic-oligosaccharides used to supplement infant formula. The latter are derived from the enzymatic cleavage of citrus pectin acidic-oligosaccharides [6]. The combination of short chain galacto-oligosaccharides/long chain fructo-oligosaccharides with pectin acidic-oligosaccharides may favour the development of a bifidogenic microbiota, decrease the growth of pathogens in the intestine, have a positive effect on the mucus layer of the gastrointestinal tract, and may stimulate the maturation of the immune system [6]–[9].

To our knowledge there are no large and longitudinal trials on the gastrointestinal tolerance and long term safety of an infant formula supplemented with the mixture comprehensive of pectin acidic-oligosaccharides in addition to the well known neutral prebiotics (short chain galacto-oligosaccharides/long chain fructo-oligosaccharides [10].

In this study, we aimed to evaluate the tolerance and safety of an infant formula supplemented with a mixture of short chain galacto-oligosaccharides, long chain fructo-oligosaccharides and pectin acidic-oligosaccharides, in healthy infants during the first year of life by investigating effects on growth, gastrointestinal tolerance and stool consistency and by identifying any adverse effects.

## Materials and Methods

The protocol of this trial and supporting CONSORT checklist are available as supporting information; see Checklist S1 and Protocol S1.

### Ethics Statement

Written informed consent was obtained from all parents before randomization. All participating centers obtained approval of their local Ethical Review Board.

### Study design

This study was performed as a double blind, placebo-controlled, randomized prospective nutritional intervention study. The study was designed to determine whether a formula supplemented with a specific mixture of neutral and acidic oligosaccharides reduces the incidence of fever episodes and the occurrence of atopic dermatitis in healthy term born infants during the first year of life [7]–[8]. The study was registered in the German Clinical Trials Register (registration code DRKS 00000201).

### Participating centres

Participating study centres were the Beatrix Children's Hospital in Groningen, the Netherlands; the Schwarzach Hospital in Schwarzach, Austria; the University Children's Hospital in Zürich, Switzerland; the Mangiagalli Hospital in Milan, Italy; the Macedonio Melloni Hospital in Milan, Italy; the Charité-Universitätsmedizin Berlin in Berlin, Germany; and the Spedali Civili Hospital in Brescia, Italy.

### Subjects

Parents of infants less than 8 weeks of age from the region of the participating centres were informed of the study and asked to contact the research centres if interested in participating.

### Inclusion criteria

We included healthy, term infants (gestational age 37 to 42 weeks), with a normal birth weight (>p10 and <p90 for gestational age according to locally applicable growth charts), aged up to 8 weeks when entering the study, without a positive family history of allergic disease (hay fever, asthma or atopic dermatitis) and without a metabolic disorder requiring a special diet.

With regard to the breast fed group additional inclusion criteria were the intention of mothers to exclusively breastfeed their infants at least for 4 months and being exclusively breastfed at time of enrollment.

### Exclusion criteria

Exclusion criteria included mothers suffering from hepatitis B, HIV or Group B streptococcal infection during pregnancy; mothers taking antibiotics during breastfeeding; infants with known congenital or postnatal diseases which could interfere with the study and any pre-study feeding of the infants which could interfere with the study.

### Procedures

Mothers were encouraged to breastfeed their infant for at least 4 months and preferably 6 months.

Enrollment and randomization occurred at the same time and were performed within 8 weeks after delivery (table 1).

**Table 1:** Baseline characteristics.

	PG	CG	BG
	N = 414	N = 416	N = 300
Age at enrollment/randomization (days)	30 (20–42)	32 (21–45)	50 (36–54)
Birth weight (grams)	3277 (2965–3610)	3320 (2930–3625)	3438 (3110–3678)
Gestational age (completed weeks)	39 (38–40)	39 (38–40)	40 (39–40)
Boys	220 (53%)	200 (48%)	134 (45%)
White race	378 (91%)	392 (94%)	287 (96%)
Vaginal delivery	261 (63%)	272 (65%)	247 (82%)
Caesarean Section (incl. Elective)	153 (37%)	144 (35%)	53 (18%)
Rooms in household (not kitchen/bathroom)	3 (3–4)	3 (3–4)	4 (3–5)
Number of children in household	2 (1–2)	2 (1–2)	2 (1–2)
Single parent family	25 (6%)	23 (6%)	17 (6%)
Furry pets in household	133 (32%)	150 (36%)	95 (32%)
Smoking mother	79 (19%)	81 (19%)	21 (7%)
Smoking father	142 (35%)	163 (40%)	81 (27%)
Education level of the mother			
primary and secondary school	206 (50%)	208 (50%)	115 (38%)
some university education/some postsecondary education/technical or trade qualification	138 (33%)	119 (29%)	107 (36%)
completed university degree	69 (17%)	89 (21%)	78 (26%)
Mother employed	252 (61%)	269 (65%)	207 (69%)
Education level of the father			
primary and secondary school	184 (46%)	188 (46%)	101 (34%)
some university education/some postsecondary education/technical or trade qualification	142 (35%)	127 (31%)	113 (38%)
completed university degree	77 (19%)	95 (23%)	85 (28%)
Father employed	369 (92%)	377 (92%)	283 (95%)

For continuous variables: median (25–75 percentile) values.

For categorical data: percentages.

PG = prebiotics group; CG = control group; BG = breastfeeding group.

Only if mothers could not or intended not to exclusively breastfeed their infants, the local study team asked mothers for their consent to participate in the study and to be randomized to one of the two formula groups.

In the case of a switch form breastfeeding to formula feeding that took place due to insufficient breastfeeding within eight weeks after delivery, mothers were also asked for their consent to participate in the study and to be randomized to one of the two formula groups.

If infants continued to assume both breast milk and at least one bottle of formula after the 8th week of age, they were allocated to the mixed feeding group.

Infants of mothers who indicated that they would exclusively breastfeed were included in the breastfeeding group. This group served as the non-randomized reference group.

Randomisation was performed stratified according to study centre. Time balanced randomisation was performed with the software RANCODE with a random permuted block size of 4. Only the hospital pharmacist had a copy of the randomisation list with the actual treatment allocation. Both the investigators and the infants' parents were blind to the group allocation. The tins and the milk powder looked and smelled identical.

Infants randomized to the prebiotics group received a regular non-hydrolysed cow's milk based formula (proteins 1,3 g/100 ml, whey/casein 60/40) complying with the EC directive on infant formulae and follow-on formulae (proteins 1,4 g/100 ml, whey/casein 50/50), to which a specific mixture of neutral short chain galacto-oligosaccharides and long chain fructo-oligosaccharides, ratio 9∶1 (IMMUNOFORTIS®, Nutricia Cuijk BV, Cuijk, The Netherlands; 85 wt%) and specific pectin acidic-oligosaccharides (15 wt%), was added.

The total amount of oligosaccharides was 8 g/L with 6.8 g/L neutral and 1.2 g/L pectin acidic-oligosaccharides. Infants randomized to the control group received a similar regular non-hydrolysed cows milk based formula without added oligosaccharides.

Study formulas were started straightaway after randomization (table 1). For both groups starter formula was provided during the first six months of life, thereafter follow-on formula was provided to all formula fed infants up to one year of age. Infants randomized to the prebiotics group continued to receive the oligosaccharides in the follow-on formula. All participating mothers from either exclusive breast feeding or formula feeding were advised not to introduce complementary foods before the age of 4 months. All study formulas were manufactured and provided by Danone Research (at the time the study was conducted NUMICO), Cuijk, The Netherlands.

### Observation period

The observation period was until the age of one year. Parents were contacted every two weeks (after the age of 16 weeks every four weeks), either by home visits, clinic visit, or phone calls. During these interviews data about the onset of gastrointestinal symptoms or any other symptoms as well as prescribed drugs were collected. To help accuracy of recall, parents kept a symptom and therapy diary.

At study entry, 2 (+/− 1 week), 4 (+/− 1 week), 6 (+/− 1 week), and 12 (+/− 2 weeks) months of life, infants were clinically examined and underwent assessment of anthropometric measurements at the study centers and their homes.

### Outcomes

#### Anthropometric measurements

The infants' anthropometric measurements (body weight, length, head circumference, mid-upper arm circumference, triceps and subscapular skinfolds) were obtained using standardized techniques [11]. The naked infants were weighed on an electronic scale accurate to 0.5 g. Recumbent length was measured on a Harpenden stadiometer to the nearest 1 mm. The head circumference was measured using a non-stretch measuring tape to the nearest 1 mm. As an indicator of body composition (regional fat and fat-free tissue), mid-upper arm circumference was measured with a non-stretch tape at the midpoint between the acromium and olecranon to the nearest 1 mm; triceps and subscapular skinfold thickness were examined by means of a Holtain skinfold caliper calibrated to 0.2 mm [12].

Skinfold thickness was assessed three times and the mean of three readings was taken. The measurements were performed by experienced personnel. Actual chronological decimal age was used to calculate the standardized anthropometric indices (z scores) [13].

The growth rate was calculated as a change in body weight from weight at study inclusion divided by the time interval from enrolment to the assessments at week 16.

#### Gastrointestinal tolerance parameters

Indicators of gastrointestinal tolerance were collected at each study point using diaries and included the following: regurgitation/reflux [spitting up (mild), posseting (moderate), vomiting (severe)], flatulence (excessive air/gas in the intestine passed through the rectum), cramps (unpleasant sensation caused by abdominal contraction), colic (intermittent attacks of abdominal pain when the baby screamed and drew up his/her legs but was well between episodes), nappy rash (redness of the skin confined to the area covered by the nappy), daily frequency of stool passage and stool consistency (on a five-point scale: 1 =  watery, 2 = loose, 3 = soft, 4 =  formed, 5 = hard).

#### Adverse events (AEs)

AEs were assessed based on inquires to the parents and on their daily records. All AEs were recorded in adverse event forms and were evaluated by the investigator for causality for the relationship to the study feeding and for severity. An AE was defined as any event, that was not consistent with the information provided in the consent form or could reasonably be expected to accompany the natural history and progression of the subject's condition throughout the study. AEs were considered as serious (SAEs) if they were fatal or life-threatening, required hospitalization or surgical intervention, resulted in persistent or significant disability/incapacity or were considered to be medically relevant by the investigator. All other AEs were categorized as non-serious. AEs were assessed according to body system.

### Power Calculation

The power of the study was calculated considering the infectious diseases as primary outcome. Starting with 500 formula infants per group, it was assumed that about 35% will be lost for the per-protocol analysis due to continuation of breastfeeding beyond the second month of life and further about 15% due to different dropout/withdrawal reasons so that 250 infants per formula group fully completing the study should be achieved. With those 250 formula infants per group completing the study without violation of the protocol a reduction of the mean number of febrile episodes by 1 which is considered as clinically relevant reduction from 4.5 infection episodes to 3.5 (SD 3.5 episodes) [home care, group care (2–6 children), and day care (>6 children)], compared to placebo can be proven with 93% power in an one-sided unpaired t-test or 89% in a two-sided unpaired t-test [14].

### Statistical Methods

All statistical analyses were performed with SAS®, Version 9 (SAS Institute,Cary, NC). A p-value of <0.05 was assumed to indicate statistical significance in all tests. All analyses were performed with stratification per centre, unless numbers were too small or a centre effect was not expected.

The analyses were performed using the intention-to-treat analysis. All infants who had been randomized in the prebiotics group or the control group were included in the analysis. As breastfeeding group infants were not randomized, no statistical analysis was performed to compare the breastfeeding group with any of the formula feeding groups.

The size of both groups had been calculated for the primary endpoint of the trial.

Continuous variables were reported as mean and standard deviation (SD) or mean and standard error of mean (SEM) or median and interquartile ranges. Categorical variables were reported as numbers or percentages. They were compared using stratified linear regression, Cochran-Mantel-Haenszel or non-parametric stratified Wilcoxon tests, as appropriate for continuous and categorical variables.

Z-scores for weight, length and head circumference were calculated with the EUROGROWTH software (http://www.euro-growth.org/).

Repeated measures linear and logistic regression, using center as a random effect, were used to compare anthropometric and gastrointestinal discomfort symptoms. The stool consistency data was analyzed using a Baseline Category Logit Model (BCL).

Compiled frequency tables for adverse events, according to the body system, were based on number of infants experiencing the (serious) adverse event. Group differences were evaluated using Fisher's exact test.

## Results

### Study population

**Figure 1:**
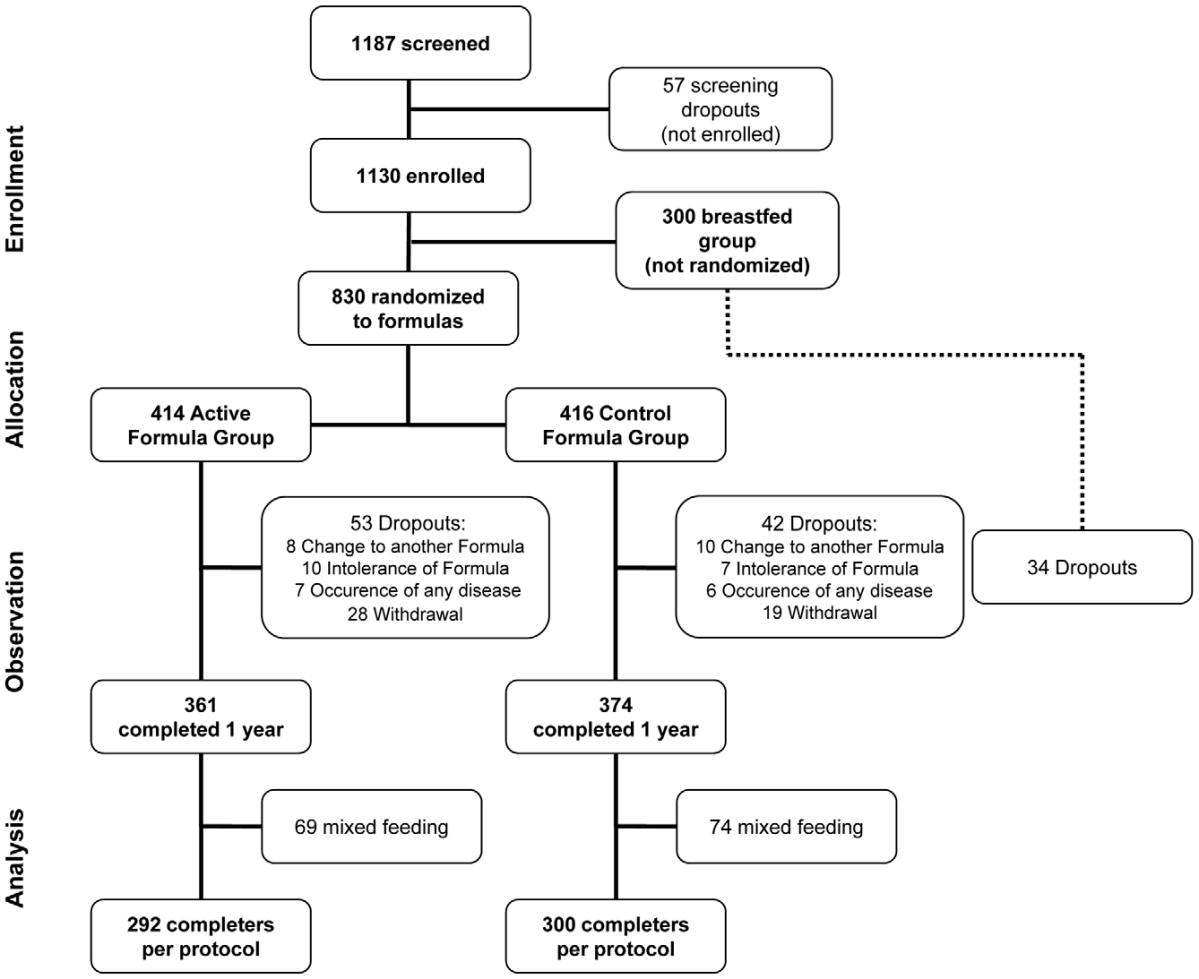
Trial profile.

From July 2005 to December 2006 a total of 1187 infants were screened. Fifty-seven of these infants were not enrolled as they did not meet the inclusion criteria. 1130 infants participated and were randomized into one of the 2 study groups or were fully breastfed and included in the breastfeeding group. The intention to treat population comprised 414 infants in the prebiotics group, 416 infants in the control group. The trial profile is shown in Figure 1. During the observation period 129 patients (11%) dropped out (prebiotics group, n = 53; control group, n = 42; breastfeeding group, n = 34). With regard to the breastfeeding group, 243 infants completed the study protocol as 23 infants switched to mixed feeding. Twenty-seven percent of the recruited infants were fully breastfed for at least 4 months. The baseline characteristics and demographics of the infants are summarized per group in Table 1. No difference between the two formula groups was found. The observation period lasted one year and ended in December 2007.

### Anthropometric measurements

There was no difference in the mean body weight, length and head circumference values between the two formula groups at any assessed time point. From enrollment to 16 weeks, the mean growth rate was 30.9 g/day with SE of 0.53 and 29.9 g/day with SE of 0.53 for the prebiotics group and for the control group, respectively.

As far as the z-scores for weight, length and head circumference are concerned, the prebiotics group and control group did not reach the range of breastfeeding group at any study point (Table 2). In the 6–12 month interval growth indices progressively declined for breastfeeding group.

**Table 2:** Weight, length and head circumference for-age z-scores.

		Weight	Length	Head circumference
8 Weeks	PG	−0,02697 (0.050)	0,04662 (0.054)	−0,0206 (0.048)
	CG	−0,00831 (0.050)	0,03694 (0.054)	−0,05479 (0.048)
	BG	0,2682 (0.060)	0,296 (0.065)	0,1318 (0.057)
16 Weeks	PG	−0,03664 (0.047)	0,08803 (0.048)	−0,03896 (0.043)
	CG	−0,00767 (0.047)	0,07352 (0.047)	−0,06434 (0.043)
	BG	0,1512 (0.056)	0,2219 (0.057)	0,05057 (0.052)
24 Weeks	PG	−0,04630 (0.045)	0,1294 (0.044)	−0,05732 (0.041)
	CG	−0,00704 (0.045)	0,1101 (0.043)	−0,0739 (0.040)
	BG	0,03428 (0.047)	0,1477 (0.045)	−0,03062 (0.045)
52 Weeks	PG	−0,08015 (0.052)	0,2744 (0.057)	−0,1216 (0.050)
	CG	−0,0048 (0.051)	0,2381 (0.056)	−0,1074 (0.049)
	BG	−0,375 (0.062)	−0,112 (0.068)	−0,3148 (0.059)

Data are presented as mean (standard error of mean).

PG = prebiotics group; CG = control group; BG = breastfeeding group.

**Figure 2:**
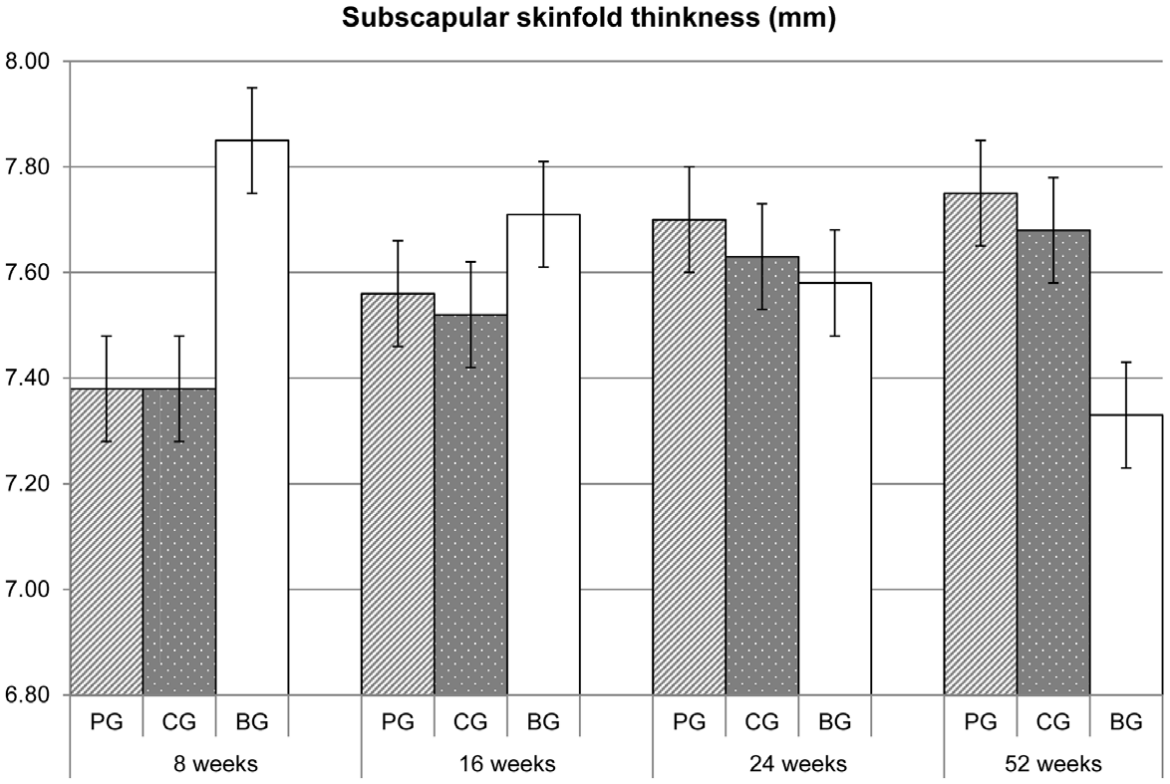
Mean (SE) subscapular skinfold thickness values. P>0.05 for comparisons between PG and CG, resulting from repeated measures linear and logistic regression, using centre as a random effect PG = prebiotics group; CG = control group; BG = breastfeeding group.

**Figure 3:**
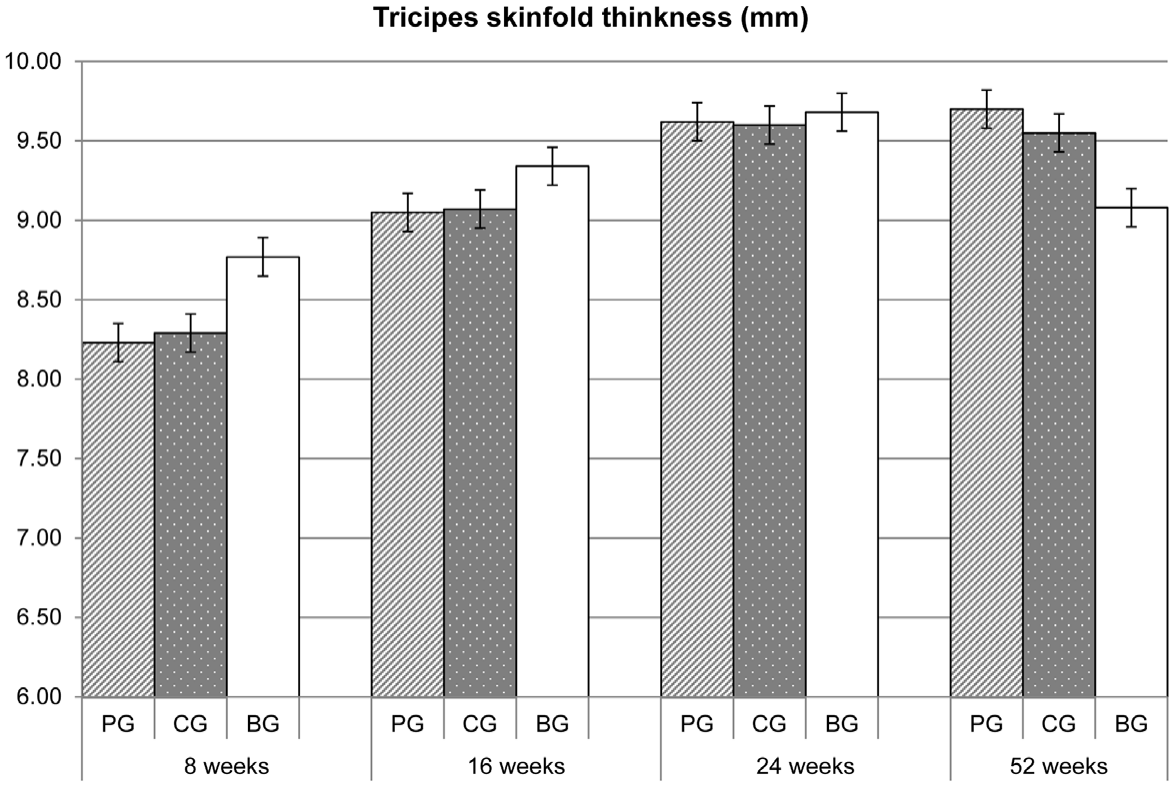
Mean (SE) triceps skinfold thickness values. P>0.05 for comparisons between PG and CG, resulting from repeated measures linear and logistic regression, using centre as a random effect PG = prebiotics group; CG = control group; BG = breastfeeding group.

No statistical significant differences were found in the skin fold thicknesses (Figure 2, 3) and in the arm circumference values (Table 3) between the two formula groups. The skin fold thicknesses assessed in the breastfeeding group at 8 weeks were strikingly larger than those in formula fed infants, whereas at 52 weeks were strikingly smaller.

**Table 3:** Arm circumference values at each study point.

Weeks	Arm circumference (cm)		
	PG	CG	BG
8	12.64 (0.06)	12.67 (0.06)	12.67 (0.07)
16	13.82 (0.05)	13.83 (0.05)	13.72 (0.07)
24	14.71 (0.06)	14.71 (0.06)	14.52 (0.07)
52	15.57 (0.06)	15.62 (0.06)	15.29 (0.08)

Data are presented as mean (standard error of mean) P>0.05 for comparisons between PG and CG, resulting from repeated measures linear and logistic regression, using center as a random effect.

PG = prebiotics group; CG = control group; BG = breastfeeding group.

### Gastrointestinal tolerance

Table 4 shows data related to the gastrointestinal symptoms during the treatment period. No difference in the incidence of any gastrointestinal symptom was detected between the two formula groups. Both formula groups showed a similar daily stool frequency at each study point**.** The incidence of the gastrointestinal symptoms and the daily stool frequency in the prebiotics group and control group partly differs from the one of breastfeeding group at any study point. No significant difference in the occurrence of nappy rash (data not shown) was found between the two formula groups. The number of spitting episodes within the first 24 weeks of age in the formula groups partly differs from the one of breastfeeding group infants, whereas the frequency of vomiting in the two formula groups was similar to that in the breastfeeding group. At 8 weeks the occurrence of colic in the prebiotics group and control group partly differs from the one of breastfeeding group. The stool frequency in the prebiotics group and control group partly differs from the one of breastfeeding group at any study point.

**Table 4:** Episodes of gastrointestinal symptoms and daily stool frequency.

		Spitting n(%)	Posseting n(%)	Vomiting n(%)	Colics n(%)	Flatulence n(%)	Cramps n(%)	Daily stool frequency mean (SD)
8 Weeks	PG	216 (56.3)	158 (41.1)	35 (9.1)	112 (27.7)	230 (56.9)	130 (32.2)	1.66 (1.282)
	CG	206 (54.4)	176 (46.4)	39 (10.3)	109 (26.4)	233 (56.4)	131 (31.7)	1.68 (1.166)
	BG	171 (67.6)	101 (39.9)	31 (12.3)	61 (21.5)	172 (60.4)	96 (33.7)	2.76 (1.945)
16 Weeks	PG	195 (51.5)	146 (38.5)	27 (7.1)	42 (10.9)	131 (33.9)	43 (11.1)	1.28 (0.750)
	CG	186 (49.1)	158 (41.7)	27 (7.1)	38 (9.7)	145 (36.9)	48 (12.2)	1.26 (0.841)
	BG	180 (65.7)	115 (42.0)	28 (10.2)	20 (7.2)	103 (36.9)	27 (9.7)	1.84 (1.280)
24 Weeks	PG	181 (47.6)	133 (35.0)	26 (6.8)	10 (2.6)	90 (23.6)	10 (2.6)	1.44 (0.743)
	CG	174 (45.8)	121 (31.8)	29 (7.6)	10 (2.6)	81 (21.0)	14 (3.6)	1.41 (0.706)
	BG	169 (62.1)	100 (36.8)	31 (11.4)	13 (4.7)	67 (24.4)	11 (4.0)	1.52 (0.911)
52 Weeks	PG	33 (9.6)	18 (5.2)	12 (3.5)	1 (0.3)	51 (14.2)	1 (0.3)	1.73 (0.779)
	CG	30 (8.4)	23 (6.4)	11 (3.1)	2 (0.5)	38 (10.2)	4 (1.1)	1.69 (0.748)
	BG	45 (17.4)	22 (8.5)	12 (4.6)	1 (0.4)	21 (7.9)	3 (1.1)	1.75 (0.869)

P>0.05 for all comparisons between PG and CG, resulting from repeated measures linear and logistic regression, using center as a random effect.

PG = prebiotics group; CG = control group; BG = breastfeeding group.

**Figure 4:**
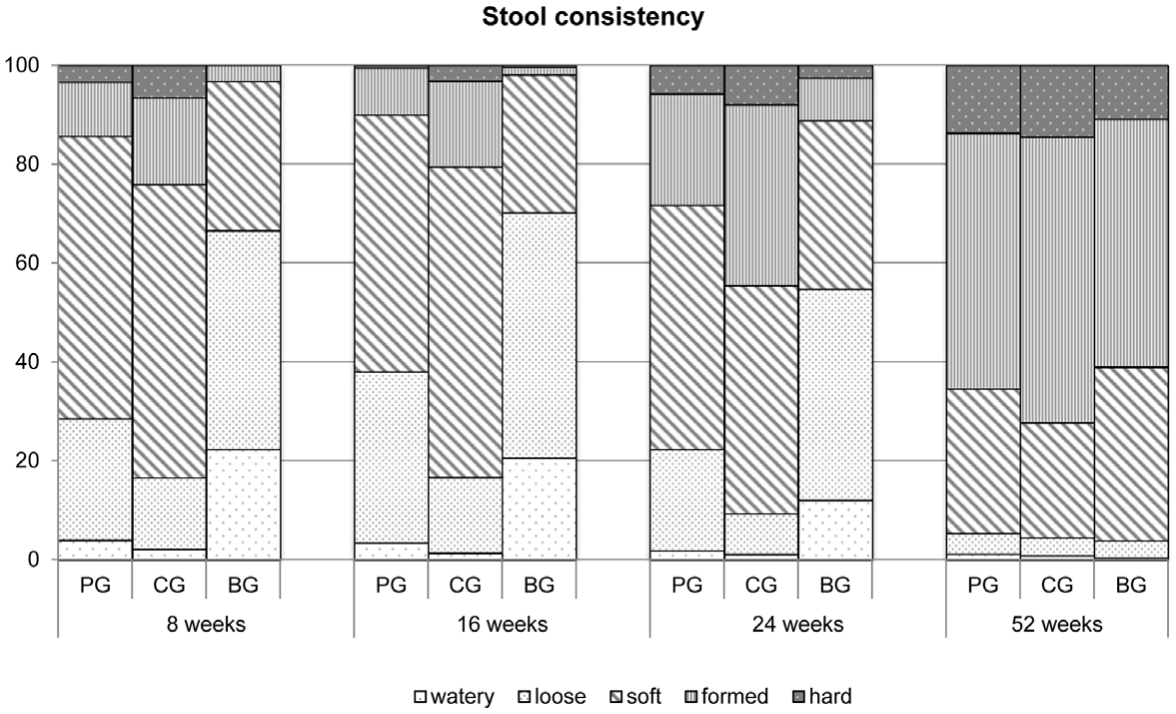
Stool consistency at each study point. P<0,05 (PG vs CG) PG = prebiotics group; CG = control group; BG = breastfeeding group.

The stool consistency was significantly lower in the prebiotics group at 8, 16 and 24 weeks as compared to the control group (p<0.001) and closer to the breastfeeding group (Figure 4). As it can be seen in table 4 the lower stool consistency score in the prebiotics group compared to the control group was associated with a higher number of loose and soft stools and a smaller number of formed and hard stools

### Adverse events

In total, 640 AEs occurred in 431 infants. Of these, 112 AEs occurring in 110 infants were assessed as serious. Documented reasons for all AEs were mostly illnesses which are common during the first year of life (i.e. otitis media, bronchitis, gastroenteritis, upper respiratory tract infection, varicella, bronchiolitis, pharyngitis, urinary tract infection). There were no differences in the incidence of AEs (prebiotics group 31% vs. control group 30%; p>0.05) and SAEs (prebiotics group 10,6% vs. control group 9,4%) between the groups. SAEs occurred in 9,3% of breast fed infants.

## Discussion

To our knowledge, the present study is the largest trial investigating the effect of supplementation with an innovative mixture of short chain galacto-oligosaccharides/long chain fructo-oligosaccharides/pectin acidic-oligosaccharides on gastrointestinal tolerance and safety in full-term infants over a period of one year. The results of this study clearly demonstrated that this prebiotic mixture containing not only the neutral oligosaccharides (short chain galacto-oligosaccharides, long chain fructo-oligosaccharides) but also the acidic oligosaccharides pectin acidic-oligosaccharides was safe and well tolerated. These results supplement the recently published efficacy data on the incidence of fever episodes and the occurrence of atopic dermatitis in healthy term born infants during the first year of life [7]–[8].

In order to assess whether the prebiotic formula is tolerated and allows a growth as found in breastfed infants, we would have had to compare the formula groups with the breastfeeding group. However, it is impossible to randomize infants to either breastfeeding or a formula. As a result, groups are not comparable and therefore we made no statistical comparisons.

The infants that received the supplemented formula showed no significant changes in z-scores for weight, length and head circumference values during the first year of life and in the rate of weight gain until the age of four months as compared to the infants fed the control formula. Furthermore, all infants exhibited linear, ponderal and head circumference growth rates within the normal range as compared with the standardized European growth references [11].

In order to exclude the possibility that the absence of any significant variation in the rate of weight gain could be due to an inadequate sample size, we performed a power calculation to detect a difference in weight gain of 3 g/day over a 3 to 4 month period, which is considered to be nutritionally significant [15], for prebiotics group versus control group in the intention-to-treat population, which was 87% (assessed by a two-sided t-test using an alpha of 5%).

As far as the breastfed infants are concerned, we found higher rates of growth increments in the first six months of life than the formula fed subjects. This finding is in agreement with results from previous studies where breastfed infants presented higher growth rate during the first months of life compared with formula-fed infants [16]–[18].

Our results are consistent with the observation that infants fed formula supplemented with different combinations of prebiotics achieve a growth within the normal range [6], [19], [20].

The effect of acidic-oligosaccharides and galacto-oligosaccharides/fructo-oligosaccharides/acidic-oligosaccharides on safety as well as tolerance was previously investigated only by Fanaro et al [6] by means of an explorative study. The authors reported that there was no difference in growth and gastrointestinal tolerance between groups. Our data confirm the results of this study conducted in a small sample of infants (n = 46) for a relatively short period of six weeks.

With regard to gastrointestinal tolerance, the occurrence of gastrointestinal symptoms was throughout the study similar in the prebiotics group and control group. In agreement with our findings, previous studies have reported that prebiotic supplementation does not affect gastrointestinal symptoms except stool consistency [19]–[22]. In our study stool consistency scores in infants fed formula supplemented with the prebiotic mixture were significantly lower than those in the control group at 8, 16, 24 weeks, and closer to those presented by breastfed infants. As seen in table 4 the lower stool consistency score in the prebiotics group compared to the control group was based on a higher number of loose and soft stools and a smaller number of formed and hard stools. This latter finding is clinically relevant, as in daily practice mothers of formula-fed infants frequently complain about high stool consistency which may cause discomfort. In addition, several studies have demonstrated that formula supplemented with prebiotics promotes the development of a stool consistency similar to those of breastfed infants [19]–[21]. Indeed, Moro et al [19] showed that short chain galacto-oligosaccharides and long chain fructo-oligosaccharides ratio 9∶1 fed to term infants reach the colon and interact quantitatively with the intestinal microbiota, imitating the fate of oligosaccharides found in human milk.

The stool frequency rate found through the study was similar in the prebiotics group and control group and it was slightly lower compared to that of breastfeeding group only in the first two months of life. Although previous studies have demonstrated that short chain galacto-oligosaccharides/long chain fructo-oligosaccharides (9∶1) determine a slight increase in stool frequency [19], [21], the tendency for increased stool frequency reported in infants fed prebiotics may not be of clinical relevance as these frequencies are in the normal range for healthy infants.

Drop-out rates, reasons for dropping out and the incidence of serious adverse events were similar in both the prebiotics group and control group demonstrating that the prebiotic formula was safe and well tolerated.

Our results demonstrate that a formula supplemented with this innovative mixture of neutral and acidic oligosaccharides is well tolerated and safe in healthy full-term infants during the first year of life.

## Supporting Information

Protocol S1 **Trial Protocol.**
(PDF)

Checklist S1 **CONSORT Checklist.**
(DOC)

## References

[1] Boehm G, Moro G (2008). Structural and functional aspects of prebiotics used in infant nutrition.. J146;Nutr.

[2] Boehm G, Stahl B, Jelinek J, Knol J, Miniello V (2005). Prebiotic carbohydrates in human milk and formulas.. Acta Paediatr.

[3] Harmsen HJ, Wildeboer-Veloo AC, Raangs GC, Wagendorp AA, Klijn N (2000). Analysis of intestinal flora development in breast-fed and formula-fed infants by using molecular identification and detection methods.. J146;Pediatr Gastroenterol Nutr.

[4] Field CJ (2005). The immunological components of human milk and their effect on immune development in infants.. J146;Nutr.

[5] Boehm G, Stahl B, Mattila-Sandholm T (2003). Oligosaccharides.. Functional dairy products.

[6] Fanaro S, Jelinek J, Stahl B, Boehm G, Kock R (2005). Acidic oligosaccharides from pectin hydrolysate as new component for infant formulae: effect on intestinal flora, stool characteristics, and pH.. J146;Pediatr Gastroenterol Nutr.

[7] van Stuijvenberg M, Eisses AM, Grüber C, Mosca F, Arslanoglu S (2011). Do prebiotics reduce the number of fever episodes in healthy children in their first year of life – a randomised controlled trial.. Br146;J146;Nutr.

[8] Grüber C, van Stuijvenberg M, Mosca F, Moro G, Chirico G (2010). Reduced occurrence of early atopic dermatitis due to immunoactive prebiotics among low atopy risk infants.. J146;Allergy Clin Immunol.

[9] Lee JH, Shim JS, Lee JS, Kim MK, Chung MS (2006). Pectin-like acidic polysaccharide from Panax ginseng with selective antiadhesive activity against pathogenic bacteria.. Carbohydr Res.

[10] Braegger C, Chmielewska A, Decsi T, Kolacek S, Mihatsch W (2010). Supplementation of Infant Formula With Probiotics and/or Prebiotics: A Systematic Review and Comment by the ESPGHAN Committee on Nutrition.. J146;Pediatr Gastroenterol Nutr.

[11] World Health Organization (2006). http://www.euro-growth.org/.

[12] Tanner JM, Whitehouse RH (1975). Revised standards for triceps and subscapular skinfolds in British children.. Arch Dis Child.

[13] Haschke F, van't Hof MA, the Euro-Growth Study Group (2000). Euro-Growth References for length, weight, and body circumferences.. J146;Pediatr Gastroenterol Nutr.

[14] Pabst HF, Spady DW, Pilarski LM, Carson MM, Beeler JA (1997). Differential modulation of the immune response by breast or formula-feeding of infants.. Acta Paediatr 1997;.

[15] U.S. Food and Drug Administration/American Academy of Pediatrics Committee on Nutrition (1988). Clinical testing of infant formulas with respect to nutritional suitability for term infants.. http://www.cfsan.fda.gov/dms/inf-clin.html.

[16] Agostoni C, Grandi F, Giannì ML, Silano M, Torcoletti M (1999). Growth patterns of breast fed and formula fed infants in the first 12 months of life: an Italian study.. Arch Dis Child.

[17] Rogers IS, Emmett PM, Golding J (1997). The growth and nutritional status of the breast-fed infant.. Early Hum.

[18] WHO Working Group on Infant Growth (1995). An evaluation of infant growth: the use and interpretation of anthropometry in infants.. Bull World Health Organ.

[19] Moro G, Minoli I, Mosca M, Fanaro S, Jelinek J (2002). Dosage-related bifidogenic effects of galacto- and fructooligosaccharides in formula-fed term infants.. J146;Pediatr Gastroenterol Nutr.

[20] Ziegler E, Vanderhoof JA, Petschow B, Mitmesser SH, Stolz SI (2007). Term infants fed formula supplemented with selected blends of prebiotics grow normally and have soft stools similar to those reported for breast-fed infants.. J146;Pediatr Gastroenterol Nutr.

[21] Boehm G, Lidestri M, Casetta P, Jelinek J, Negretti F (2002). Supplementation of a bovine milk formula with an oligosaccharide mixture increases counts of faecal bifidobacteria in preterm infants.. Arch Dis Child Fetal Neonatal Ed.

[22] Rao S, Srinivasjois R, Patole S (2009). Prebiotic Supplementation in Full-term Neonates.A146;Systematic Review of Randomized Controlled Trials Arch Pediatr Adolesc Med..

