# Supplementary material for: Tolerance and Safety Evaluation in a Large Cohort of Healthy Infants Fed an Innovative Prebiotic Formula: A Randomized Controlled Trial
Source: PLoS One. 2011 Nov 30;6(11):e28010. doi: 10.1371/journal.pone.0028010 (PMC3227609; doi:10.1371/journal.pone.0028010)
Supplement: Protocol S1 — Trial Protocol. (PDF) [file pone.0028010.s001.pdf]

Lower incidence of infection

by formulas supplemented with a mixture of immunologically active neutral and acidic oligosaccharides

Numico multicentric infection prevention study

C O N F I D E N T I A L

Protocol Number: NRG / II / DbCoRPMc / IC Inf 01

Protocol Status: FINAL

Preparation Date: June 2nd 2005 (JJ)

Double-blind, controlled and randomised study with a parallel group design on the effect of formula feeding (IF and FOF) supplemented with a mixture of immunologically active neutral and acidic oligosaccharides on the incidence of febrile respiratory and gastrointestinal infections in healthy term born infants during the first year of life

Confidentiality Statement
The information contained in this document, especially unpublished data, is the property of NUMICO Research, and is therefore provided to you in confidence as an investigator, for review by you, your staff and an applicable Ethics Review Committee. It is understood that this information will not be disclosed to others without written authorisation from NUMICO Research Germany, except to the extent necessary to obtain informed consent from those persons to whom the product may be administered.

Study Supervisor, University of Berlin:
Prof. Dr. U. Wahn
Virchow University Hospitals
Medical Faculty of the Humboldt University Berlin, Childrens Hospital
Heubnerweg 6, D-14059, Berlin, Germany
Phone: ++49-30-450-566182, Fax: ++49-30-450-566931, Email: marina.birr@charite.de

Study Leader, University of Groningen:
Prof. Dr. P.J.J. Sauer
Academisch Ziekenhuis Groningen / Groningen University Hospital
Hanzeplein 1, NL-9700 RB Groningen, The Netherlands
Phone: ++31-50-3612470, Email: p.j.j.sauer@bkk.azg.nl

Study Leader, University of Milan:
Prof. Dr. F. Mosca
Division of Neonatology and Neonatal Pathology, I.C.P.
Mangiagalli Hospital, Via Commenda 12, I-20123 Milan, Italy
Phone: ++39-02-57992499, Fax: ++39-02-1382-57992429, Email: fabio.mosca@neonatologia-icp.it

Study Leader, University of Naples:
Prof. Dr. A. Guarino
Department of Pediatrics, University "Federico II", Via S. Pansini 5, I-80131 Naples, Italy
Phone: ++39-081-7464235, Fax: ++39-081-5451278, Email: alfguari@unina.it

Study Leader, University of Zurich:
Prof. Dr. C.P. Braegger
Division of Gastroenterology and Nutrition
Children's University Hospital, Steinwiesstr. 75, CH-8032 Zurich
Phone: ++49-12-667323, Fax: ++49-12-667171, Email: christian.braegger@kispi.unizh.ch

Study Manager, NUMICO Research Germany:
Dr. Jürgen Jelinek
NUMICO Research Germany, Bahnstraβe 14-30, D-61381 Friedrichsdorf, Germany
Phone: ++49-6172-991838, Fax: ++49-6172-991862, Email: juergen.jelinek@milupa.de

Co-ordination for NUMICO Research Germany:

Bahnstrasse 14-30
D-61381 Friedrichsdorf/Ts. / Germany
Phone: ++49-6172-991321
Fax: ++49-6172-991862
Email: milupa.research@t-online.de

Dr. Günther Boehm
Director Infant Nutrition Research
Phone: ++49-6172-991320
Email: guenther.boehm@milupa.de

Dr. Jürgen Jelinek
Head Data & Information Management
Phone: ++49-6172-991838
Email: juergen.jelinek@milupa.de

Dr. Bernd Stahl
Head Carbohydrate Research
Phone: ++49-6172-991496
Email: bernd.stahl@milupa.de

Anja Frings
Clinical Trial Researcher
Phone: ++49-6172-991322
Email: anja.frings@milupa.de

PROTOCOL SYNOPSIS

TITLE	Double-blind, controlled and randomised study with parallel group design on the effect of formula feeding (IF/FOF) supplemented with a mixture of immunologically active neutral and acidic oligosaccharides on the incidence of febrile respiratory and gastrointestinal infections in healthy term born infants during the first year of life
SPONSOR	Milupa GmbH - NUMICO Research Germany
OBJECTIVES	The study will elucidate whether formula feeding (IF/FOF) supplemented with a mixture of immunomodulating neutral and acidic oligosaccharides is able to reduce the incidence of infectious illness in healthy term born infants during the first year of life as indicated by the number of febrile episodes and symptoms of infection.
STUDY DESIGN	Nutritional intervention in healthy term born infants with IF and FOF in a double-blind, placebo-controlled, randomised, prospective study with a parallel group design till the infants' age of one year.
PLANNED SAMPLE SIZE	About 250 completers per group. Assuming a withdrawal/dropout rate of 15% during the study duration and a loss of completers for the per-protocol (PP) analysis of 35% the samples size needed to start with is about 500 per group (ITT with all).
SUBJECT SELECTION CRITERIA	Healthy, term infants (GA 37-42 weeks), normal birth weight (girls: 2.7-5.0 kg, boys: 2.9-5.2 kg), no high risk to develop an atopic disease and no metabolic disorders requiring a special diet other than standard non-hydrolyzed IF/FOF
TEST GROUPS	Group 1: Active Formula Group with babies on full formula feeding after 2 months at the latest receiving a non-hydrolysed cow's milk based formula containing an immunomodulating mix of neutral and acidic oligosaccharides. To avoid any immunological influence by other ingredients, the formula will not contain any other special supplementation such as probiotics, LCPUFA, or nucleotides.<br>Group 2: Control Group with babies on full formula feeding after 2 months at the latest receiving as placebo a standard non-hydrolysed cow's milk based formula with the same composition as the active formula but without supplementation of oligosaccharides.<br>Reference: Infants on full breastfeeding for at least 4 months.
MAIN PARAMETERS OF EFFICACY	Primary parameter of the study is the number of febrile episodes with a peak rectal temperature of at least 38.5°C in healthy term born infants during the first year of life.<br>Secondary parameters are<br>a) symptoms, diagnoses and severity of infectious illness, e.g.<br>• duration (days) of symptoms<br>• duration (days) on medical treatment (paracetamol, antibiotics, etc.)<br>• number of visits to hospital/practitioner<br>• absence from day-care due to illness<br>• hospitalisation days, etc.<br>b) biochemical plasma parameters of immune status, at least in a subgroup of infants, including response to vaccination, e.g.<br>• antibodies<br>• lymphocyte subpopulations<br>• cytokines<br>c) stool parameters, at least in a subgroup of infants<br>• CFU/g stools of Bifidus sp. and Lactobacilli sp.<br>• presence of pathogens measured as CFU/g stools<br>• faecal pH<br>• faecal sIgA<br>d) occurrence of allergic symptoms (atopic dermatitis)

MAIN PARAMETERS OF SAFETY	Absence of unanticipated adverse events with special focus on<br>• Incidence of gastrointestinal discomfort<br>• Anthropometric parameters<br>• Acceptance and tolerance
STATISTICAL ANALYSIS	ANOVA/T-tests for parametric group tests of interval data; Kruskal-Wallis/U-tests for non-parametric group tests of interval data and ordinal data; Chi^2-/Fisher's exact tests for qualitative (categorical and nominal) data; Kaplan-Meyer-curves/Log-rank tests for time-related data; multi-variate analyses, regressions, logistic regressions for relationships between and interactions of parameters
STUDY OUTLINE	Mothers and their infants from the region of Berlin, Groningen, Milan, Naples and Zurich will be approached in the first 8 weeks of the infant's age to participate in the study if they fulfil all inclusion but not exclusion criteria. Participation in the study will be voluntary and written informed consent will be obtained.<br>Mothers who cannot/do not fully breastfeed their infants and have fed their infant at least one bottle of standard (non-hydrolysed) cow's milk based formula will be asked to be randomised to one of the two formula groups. The formulas (IF/ FOF) will be used until the infants reach 1 year of age. Mothers fully breastfeeding their infant at study entry are asked to join the reference group.<br>At inclusion (examination day 1), questionnaires will be filled in with data on the mother incl. age, demography and socio-economic status, occupation, general health, nutritional status, delivery etc. Furthermore, the infants will be clinically examined and data on their feeding pattern and health status collected (basal questionnaire). A stool sample will be taken before the study feeding starts.<br>The mothers will be issued with a diary supporting the collection of data on feeding pattern, occurrence of any symptoms of illness etc. afterwards.<br>There are 4 further examination days planned with personal contacts (visits at clinic or at home) at the infant's age 2, 4, 6, and 12 months, at which the clinical examinations incl. anthropometry will be repeated and again data on feeding pattern and health status (follow-on questionnaire) as well as a stool sample will be collected. Acceptance and tolerance of the study feeding regimens as well the occurrence of allergic symptoms (atopic dermatitis) will also be checked.<br>If the mother gave her written informed consent at study entry that the child may provide two blood samples (2-3 ml), i.e. at the infant's age of 6 and 12 months, a respective blood sample will be collected at the examination days 4 and 5.<br>In between the examination days with personal contacts to the mothers and their infants, there are structured interviews planned, during which the parents are asked about the health status of their infant (occurrence of fever, other symptoms of illness, use of medication etc.), the feeding pattern incl. acceptance and tolerance, possible complaints, etc. (follow-on questionnaire).<br>The combination of 5 major visits in the clinic/at home and regular intermittent contacts (home visits, telephone calls) will guarantee contacts to the mothers in 2-4 weekly intervals (2 weekly till the infant's age of 4 months, 4 weekly afterwards). This will enhance study adherence and allows the cross-check of the questionnaires.<br>If any illness of the infants will require a visit to the clinics/ practitioners, illness related details should also be collected for later evaluation (diagnoses, medication etc.).
START STUDY	Start of recruitment in June (1st center) – August 2005 (last center)
END STUDY	End of clinical phase in July 2007 (last center)

TABLE OF CONTENTS Page

1.	Agreement and signatures	8
2.	Introduction	9
3.	Extended Rationale	10
3.1	Prebiotics: Neutral oligosaccharides (GOS/FOS)	11
3.2	Acidic oligosaccharides (AOS)	12
3.3	Experimental results	14
3.4	Clinical results	14
4.	Aim of the study	15
5.	Working hypothesis	15
6.	Study parameters	16
6.1	Primary outcome parameters	16
6.2	Secondary outcome parameters	16
6.3	Safety parameters	17
6.4	Possible confounding parameters	18
7.	Study design	19
8.	Study duration	19
9.	Study groups	19
10.	Study subjects	20
10.1	Enrolment of subjects	20
10.2	Inclusion criteria	21
10.3	Exclusion criteria	21
10.4	Randomisation	21
10.5	Number of subjects and study power	21
10.6	Compensation to study subjects	22
11.	Study test products	23
11.1	Basic product composition and packaging / blinding	23
11.2	Preparation, administration and dosage	23
11.3	Assessment of feeding compliance	24
11.4	Logistics of product supply	24
11.5	Additional feeding recommendations	24
12.	Study proceeding in detail	25
12.1	Enrolment	25
12.2	Study groups	25
12.3	Per-protocol- and intention-to-treat-analysis	25
12.4	Examination day 1	26
12.5	Additional examinations in study subgroups	26
12.6	Interviews	27
12.7	Further examination days	27
12.8	Vaccination / vaccination response	27

TABLE OF CONTENTS (continued) Page

12.9 Special recheck of feeding group 27
12.10 General assessment of each study subject during and after the study 28
12.11 Provision of formulas 28
12.12 Study schedule overview 28

13. Data and sample collection and analysis 30
13.1 Number of febrile episodes (primary outcome parameter) 30
13.2 Symptoms of illness (secondary outcome parameter) 30
13.3 Faecal parameters (secondary outcome parameter) 30
13.4 Plasma parameters (secondary outcome parameter) 30
13.5 Allergy symptoms (secondary outcome parameter) 31
13.6 Anthropometrics and other safety parameters 31

14. Statistical analysis 33
14.1 Basic statistical procedure 33
14.2 Descriptive analyses 34
14.3 Comparative analyses 34
14.3.1 Interval data 35
14.3.2 Ordinal data 35
14.3.3 Nominal data 35
14.4 Correlations 35

15. Documentation 36

16. Clinical monitoring 37

17. Confidence and publication 37

18. Withdrawals and dropouts 37

19. Personal responsibilities 39
19.1 Responsibility of the participating clinicians 39
19.2 Responsibility of NUMICO Research Germany 40
19.3 Schedule of responsibilities 41

20. Protocol changes 41

21. Appendix 42
21.1 Composition of study formulas 42
21.2 Multi-language labels of study formulas 43
21.3 Guidelines on dosage and preparation of study infant milk formulas 44
21.4 Time-balanced randomization tables and recording of the enrolment dates 46
21.5 Example of a sealed envelope for unblinding of a study subject 48
21.6 Letter to the parents for information about the study 49
21.7 Written consent of the parents for participation of their infant in the study 52
21.8 Study insurance documents 54
21.9 Case report forms and diaries 54

1. AGREEMENT AND SIGNATURES

This document is a confidential communication of NUMICO Research Germany. The recipient agrees that no unpublished information contained herein will be published or disclosed without NUMICO Research Germany prior written approval. However, this document may be disclosed to appropriate Ethical Review Committees or Institutional Review Boards or duly authorised representatives of a national regulatory authority under the condition that they are requested to respect confidentiality.

The signatures of the principal investigator and operators below constitute their agreement to this protocol and provide the necessary assurance that:

1. this study will be conducted according to all stipulations of the protocol as specified in both the clinical and administrative sections, and

2. all statements regarding confidentiality will be complied with.

---	-----------------------
Prof. Dr. U. Wahn, Study Supervisor, University of Berlin	Date
---	-----------------------
Prof. Dr. P.J.J. Sauer, Study Leader, University of Groningen	Date
---	-----------------------
Prof. Dr. F. Mosca, Study Leader, University of Milan	Date
---	-----------------------
Prof. Dr. A. Guarino, Study Leader, University of Naples	Date
---	-----------------------
Prof. Dr. C.P. Braegger, Study Leader, University of Zurich	Date
---	-----------------------
Dr. G. Boehm, Director Infant Nutrition Research, Numico	Date
---	-----------------------
Dr. J. Jelinek, Study Manager, Numico Research Germany	Date
---	-----------------------
Dr. B. Stahl, Project Leader, Numico Research Germany	Date

2. INTRODUCTION

Breast milk is a living substance containing nutrients, hormones and other biologically active constituents which aid appropriate growth and development (Hambreus, 1977; Inch, 1996). There is clear evidence for major health benefits for both mother and baby. In infancy, breastfeeding has a protective effect, e.g. against gastrointestinal and respiratory infections, middle ear and urinary tract infections, neonatal septicemia and NEC (Howie PW et al: Protective effect of breast feeding against infection. Br Med J 1990; 300:11-16; Wold AE & Adlerberth I: Breast feeding and the intestinal microflora of the infants – implications for protection against infection diseases. Adv Exp Med Biol 2000; 478:77-93; Pisacane A et al: Breast-feeding and urinary tract infections; J Pediatr 1992; 120(1):87-89; Duncan B et al: Exclusive breastfeeding for at least 4 months protects against otitis media. Pediatrics 1993; 95:867-872; Dewey KG et al: Differences in morbidity between breast-fed and formula-fed infants. Pediatr 1995; 126:696-702; Lucas A, Cole TJ: Breast milk and necrotising enterocolitis. Lancet 1990; 336:1519-1523).

Breast milk is known to provide advantages to the incompletely developed neonatal immunity and thus also increases active immune response to immunisation, enhancing its benefits (Hahn-Zoric M et al: Antibody responses to parenteral and oral vaccines are impaired by conventional and low-protein formulas as compared to breastfeeding. Acta Paediatr Scand 1990; 79:1137-1142; Pabst HF et al: Differential modulation of the immune response by breast or formula-feeding of infants. Acta Paediatr 1997; 86:1291-1297).

Due to important differences between the composition of breast milk and infant formulas, epidemiological evidence indicates that breastfed infants are at significantly reduced risk of infection even in industrialised societies. The protective effect of breast feeding against infectious diseases is provided by specific (antibody dependent) and broad, non-specific protective factors in human milk (oligosaccharides, proteins, glycoproteins, and lipids).

To simulate this functionality with the aim to mimic the immune-mediated beneficial effect of breast milk on morbidity and mortality of newborn infants, Numico has developed a mixture of immunologically active neutral and acidic oligosaccharides for a balanced maturation of the gastrointestinal tract and the immune system.

3. EXTENDED RATIONALE

Newborn babies and young infants are especially vulnerable for infectious diseases. This is mainly due to their immature GI-tract as well as based on their not yet fully developed immune system after birth. Since several years there is an increasing evidence that the cross link between the GI tract (barrier function, intestinal flora) with its effects on the epithelial cells and the underlying gut-associated lymphoid tissue (GALT) is of importance. The GALT is one of the body's largest subparts of the immune system. Due to the specific situation during the fetus development in utero the immune system of mother and child is biased to allow for growth and development of the fetus but not reacting against the immunological incompatible fetal tissue expressing both maternal and paternal antigens. This situation can be classified in a simplified manner by focussing on the specific/adaptive part of the immune system. A specific subset of the T-lymphocytes the so called T-helper cells are an important constituent of the specific immune system. The T-cells can be divided into TH1, TH2, TH3 subpopulations based on their effector functions and cytokine expression.

Bacterial and viral infections induce a TH1 dominant response and TH2 cells are involved in regulation of the IgE production as well as in inflammation and later on the bronchiolar hyper reactivity in asthma. TH3 are involved in the tolerance induction for the recognition self/non-self. Especially the balance between TH1- and TH2-helper cells can be used to classify the status of the immune system.

Newborn infants are born with a biased to TH2. This is required until birth to suppress cytolytic reactions between mother and fetus.[1] [2] Directly after birth the adaptive but also the innate immune system has to be further developed to prevent infection and other diseases. In healthy-born and especially breast fed infants the immune system develops to a balanced TH1 and TH2 system. Strong imbalances in direction of TH1 are associated with autoimmune diseases those in the direction of TH2 with increases incidences of atopic diseases, allergy and asthma. Especially the imbalance in direction of TH2 is relevant due to the steadily increase of atopic diseases and allergies over the last decades.

The postnatal period is a crucial time for the maturation of the immune system. At birth T-lymphocytes exhibit a TH2-profile, characterized by a limited ability to produce cytokines. Throughout the first months after birth these TH2-skewed responses are modified into low-level immunity, predominantly expressing TH1-cytokines and IgG-antibodies, particularly of the IgG1 subclass.[3] [4]

In breast fed infants the maturation of the gut and immune system is perfectly supported by the concerted action of active compounds of mother's milk. The main fac-

---

[1] Langrish CL, Buddle JC, Thrasher AJ, Goldblatt D. Neonatal dendritic cells are intrinsically biased against Th1 immune responses.Clin Exp Immunol 2002; 128:118-123

[2] Morein B, Abusugra I, Blomqvist G. Immunity in neonates Vet Immunol Immunopathol 2002; 87:207-213

[3] Holt PG, Jones CA. The development of the immune system during pregnancy and early life. Allergy 2000; 55:688-697

[4] Prescott SL, Macaubas C, Smallacombe T, Holt BJ, Sly PD, Holt PG. Development of allergen-specific T-cell memory in atopic and normal children. Lancet 1999; 353:196-200

tors are oligosaccharides (prebiotic, anti-infective and immune modulative), lipids (PUFA for membrane development, metabolites for inflammation processes, barrier function), and proteins (balanced source of amino acids, manifold immunoactive agents such as immunoglobulins, growth factors) and nucleotides. Based on recent research activities on human milk a new concept was created to develop an infant formula (IF and FOF) following partly the functional complexity of mother's milk.

3.1 Prebiotics: Neutral oligosaccharides (GOS/FOS)

Prebiotics are necessary for the development of a *Bifidus-dominant flora* and overall breast-fed-type flora in the large intestine. This function of human milk oligosaccharides is resembled by the prebiotic mixture of short chain GOS and long chain FOS.[5] [6] [7] [8] The pattern of subspecies of bifidobacteria in the prebiotics group is highly comparable to the pattern found in breast-fed infants.[9] In context with allergies Ouwehand et al found that allergic infants have a Bifidus subspecies pattern of an adult like flora.[10]

The *stool characteristics* measured as frequency and consistency are significantly changed to a breast-fed like type (more frequent and softer) by oligosaccharides to characteristics found in babies fed human milk.

GOS/FOS significantly lowered the *stool pH* in the direction of breast fed infants which results in an antibacterial environment and thus contributes to the suppression of pathogens in the infantile gut.[7]
GOS/FOS not only stimulates the growth of bifidobacteria but also reduce the absolute number as well as the proportion of clinically relevant *pathogens* in the intestine which could reduce the risk of infections.[11]

---

[5] Rigo J, Pieltain C, Studzinski F, Knol J, Bindels JG. Clinical evaluation in term infants of a new formula based on prebiotics, β-palmitate and hydrolysed proteins. J Pediatr Gastroenterol Nutr 2001; 32:402

[6] Boehm G, Lidestri M, Casetta P, Jelinek J, Negretti F, Stahl B, Marini A. Supplementation of an oligosaccharide mixture to a bovine milk formula increases counts of faecal bifidobacteria in preterm infants. Arch Dis Child 2002; 86(3):F178-181

[7] Moro G, Minoli I, Mosca M, Fanaro S, Jelinek J, Stahl B, Boehm G. Dosage related bifidogenic effects of galacto- and fructo-oligosaccharides in formula-fed term infants. J Pediatr Gastroenterol Nutr 2002; 34:291-295

[8] Schmelzle H, Wirth S, Skopnik H, Radke M, Knol J, Bockler HM, Bronstrup A, Wells J, Fusch C. Randomized double-blind study of the nutritional efficacy and bifidogenicity of a new infant formula containing partially hydrolyzed protein, a high beta-palmitic acid level, and nondigestible oligosaccharides. J Pediatr Gastroenterol Nutr 2003; 36:343-351

[9] Knol J, Steenbakkers GMA, van der Linde EGM et al. Bifidobacterial species that are present in breast fed infants are stimulated in formula fed infants by changing to a formula containing prebiotics. JPGN 2002; 34:477

[10] Ouwehand AC, Isolauri E, He F, Hashimoto H, Benno Y, Salminen S. Differences in Bifidobacterium flora composition in allergic and healthy infants. J Allergy Clin Immunol. 2001; 108:144-145

[11] Boehm G, Lidestri M, Casetta P, Jelinek J, Knol J, Negretti F, Stahl B, Marini A. Effect of increasing number of intestinal bifidobacteria on the presence of clinically relevant pathogens. J Pediatr Gastroenterol Nutr 2003; 36:578

Short chain fatty acids (SCFA) are produced as a result of fermentation of GOS/ FOS in the large intestine. The intestinal SCFA pattern was characterised by a high percentage of acetate and a low percentage of both butyrate and propionate, thus mimicking the SCFA pattern of breast-fed infants. An increased level of acetate is thought to help promote the development of the protective mucous layer lining the GI tract, thereby reducing the ingress of micro organisms and food antigens which is likely to result in less infections and allergies.[9 12]

First data of a clinical study indicate that the *calcium absorption* might be positively influenced by GOS/FOS.[13] A corresponding calcium balance study has been started.

3.2 Acidic oligosaccharides (AOS)

Especially the so called acidic oligosaccharides of human milk showed in several experimental studies *an anti-adhesive (i.e. anti-infective) effect* against pathogens by resembling the soluble analogues of the host cell surface glycoconjugates (glycoproteins/glycolipids).[14] These complex molecules have a negative charge due to sialic acid as one of the monosaccharide components. Their amount is in the range of 1/10 to 1/4 of the total amount of human milk oligosaccharides.

Carrot soup is used in the therapy of diarrhoea disease since more than 100 years[15] caused by the pathological adherence of enterobacteria on the intestinal epithelia carrot soup used today in clinical treatment helps to consolidate the stool of the children, to shorten the convalescence time.[16] The carrot soup is used as well for healing dyspepsies.[17]
Guggenbichler et al. showed that the carrot soup substances, galacturonic acid, which are extracted by cooking, have the activity to block the adherence of different E. coli strains to human uroepithelial cells.[18]

---

[12] Willemsen LE, Koetsier MA, van Deventer SJ, van Tol EA. Short chain fatty acids stimulate epithelial mucin 2 expression through differential effects on prostaglandin E(1) and E(2) production by intestinal myofibroblasts. Gut. 2003; 52:1442-1447

[13] Lidestri M, Agosti M, Marini A, Boehm G. Oligosaccharides might stimulate calcium absorption in formula-fed preterm infants. Acta Paediatr 2003; 92 Suppl 441:91-92

[14] Boehm G, Stahl B. In: Mattila-Sandholm T (ed): Functional dairy products. Woodhead Publ Ltd 2002; pp203-243

[15] E. Moro. München. Med Wschr 1908; 31:1637-1640

[16] Haschke F, Tsarmaklis G, Steffan I, Heresch F. Analyse der Karottensuppe. Wiener klinische Wochenschrift 1980, Heft 2

[17] Hottinger A. Die Ernährung des Säuglings. Lehrbuch der Pediatrie. Fanconi 1967; pp112-128

[18] Guggenbichler JP, De Bettignies-Dutz A, Meissner P, Schellmoser S, Jurenitsch J. Acidic oligosaccharides from natural sources block adherence of E. coli on uroepithelia cells. Pharmceutical and Pharmacological Letters 1997; 1:35-38

Investigations into processing of carrots showed that heating-induced softening is accompanied by changes in the solubility, size and charge density of pectin polysaccharides, as a result of β-eliminative degradation.[19 20]

Analogue to the cooked carrot soup a pectin hydrolysate by enzymatic treatment of citrus pectin has been produced. Sodium hydroxide, pectolytic enzymes and demineralised water are used as processing aids. The quality of all raw material and processing aids meet food regulations.

The resulting citrus pectin digest has the nearly identical size and structures (degree of methylation, double bonds) as the carrot soup. The microbiological analysis of pectin hydrolysate is good.[21]

<u>Further references on Pectin/AOS/Carrots</u>

Maturation of small intestine in rats (villus heights▲, crypt depth ▲)
Andoh A et al. JPEN. 1999; 23:S70-S73

Stimulate IgA and IgG production in rats (spleen and MLN)
Yamada et al. Biosci Biotechnol Biochem 1999; 63:2163-2167
Yamada et al. Biosci Biotechnol Biochem 2003; 67:429-433

Stimulate IgA ▲, IgE ▼ and reduced IL4/IL10 ▼ in mouse (MLN: Th1 ▲)
Lim BO et al. Biosci Biotechnol Biochem 2003; 67:1706-1712

Inhibition of allergic asthmatic response (OVA Balb/c mice)
Lee JC et al. J Altern Compl Med 2004; 10:527-534

Modulate intestinal immune function in rats (serum: IgE ▼, IgA ▲, IgG ▲)
Lim BO et al. Nutr 1997; 127:663-667

Carrot extracts with antimicrobial function on bacteria and yeasts
Babic et al. J Appl Bacteriol 1994; 76:135-141
Nguyen-The C, Lund BM. Bacteriol 1991; 70:479-488

Carrots on immune function in rats (TH1/TH2 balance, IgE ▼, anaphyl. resp. ▼)
Akiyama H et al. Biol Pharm Bull 1999; 22:551-555

[19] Ng A, Waldron KW. Effect of cooking and pre-cooking on cell-wall chemistry in relation to firmness of carrot tissues. Journal Science, Food and Agriculture 1997; 73:503-512

[20] Doesburg JJ. Relation between the behaviour of pectic substances and changes in firmness of horticultural products during heating, Qualitas plantarum et materiae vegitables 1961; 8:115

[21] CLF Analytical Report DENFO0347509TAS1

3.3 Experimental results

The experimental results with the single active ingredients are listed under main function in the table below. First data show that a combination of GOS/FOS with AOS may have an additive/synergistic effect in animal trials on balance of the TH1/TH2 system.

Ingredient	Source	Main Function	Concentration / 100 ml	Composition
GOS/FOS	Lactose / Chicoree	TH-Cell-Balance, IgE reduction<br>Total Gut Flora<br>Promotion of Bifidus<br>Anti-Pathogen	0.68 g	GOS:FOS = 9:1
Acidic OS	(Citrus) Pectin	TH-Cell-Balance<br>Gut Barrier<br>Reduction of pH<br>Anti-Pathogen	0.12 g	Pectin-Oligosaccharides

3.4 Clinical results

Beside the various studies with GOS/FOS cited in the text under para 3.1 first interim data indicate that GOS/FOS have in addition to the *anti-pathogenic* effect also an *anti-infective* effect. In a first pilot trial it could be shown that the number of episodes of respiratory infections in the postnatal period was decreased in group of healthy term infants fed a GOS/FOS supplemented formula when compared to a group fed standard starting formula.[22]

In term infants at risk for allergies, first data indicate also an effect of GOS/FOS on the immune system with regard to *reduction of IgE levels* in the blood.[23] IgE is strictly related to allergy.

For AOS, a first study on the nutritional efficacy was performed in healthy term born infants (n=15 per group) during the first two months of life with AOS concentrations similar to those found in human milk. AOS as isolated supplement or in combination with GOS/FOS was well tolerated and no indication of a group difference for crying, regurgitation or vomiting was found. In no case any side effect occurred. Weight and length gain was normal in all infants. Significant prebiotic effects on stool pH and stool consistency were observed in the AOS group compared to the control group, however, the effect was not as strong than observed for the group fed the formula supplemented with the combination of GOS/FOS and AOS. As there was no effect of the AOS on the counts of bifidobacteria in the stools, a prebiotic effect different from a bifidogenic effect is indicated.[24]

[22] Bruzzese B, Guarino A et al. Quaderni di Pediatria 2004; 3:98
[23] Moro G et al., presented at the 21st Int Symp Neonat Int Care Milano, Nov 2004
[24] Fanaro S, Jelinek J, Stahl B, Boehm G, Vigi V. Submitted to J Pediatr Gastroenterol Nutr

Also in another study (publication in preparation) with a higher number of healthy term infants (n=27 per group) at an age of 1 week up to 3 months at recruitment and an observation period of about 4 months, all infants grew similar when being fed a formula supplemented with GOS/FOS or a combination of GOS/FOS and AOS compared to a non-supplemented control group (due to the different age at study entry infants were followed up by calculation of z-scores from the Eurogrowth study to adjust for age and gender). Also for the other parameters investigated, e.g. stool consistency, occurrence of diarrhoea, regurgitation or other health complaints no peculiarities occurred and in no case a group difference could be observed.

4. AIM OF THE STUDY

The aim of the study is to investigate whether formula feeding (IF/FOF) supplemented with a mixture of immunomodulating neutral and acidic oligosaccharides is able to reduce the incidence of infectious illness in healthy term born infants during the first year of life.

5. WORKING HYPOTHESIS

It is hypothesised that dietary intervention with a formula (IF/FOF) supplemented with a mixture of immunomodulating neutral and acidic oligosaccharides will significantly reduce the incidence of febrile infectious diseases in healthy term born infants during the first year of life when compared to control infants receiving the placebo formula as indicated by

- primary parameter: number of febrile episodes (peak rectal temperature at least 38.5 °C) and
- secondary parameter: symptoms of infection (cough, wheeze, runny nose, vomiting, diarrhoea etc.) and their duration.

6. STUDY PARAMETERS

6.1 Primary outcome parameter

The primary outcome parameter is the number of febrile episodes (peak rectal temperature at least 38.5 °C) during the first year of life.

The febrile episode is considered as finished if the child is again without fever and other symptoms. Another febrile episode is considered as new period if the fever occurs at least three days after the stated time of recovery from a previous episode.

6.2 Secondary outcome parameters

As secondary outcome parameters symptoms of illnesses indicating a viral or bacterial infection (cough, wheeze, runny nose, vomiting, diarrhoea, others) as recorded in the diaries of the mothers.

The definition of such symptoms is as follows

- *Cough:* sudden expulsion of air from the lungs with a noise that clears the air passages
- *Wheeze:* breathing with an abnormal (e.g. husky or whistling) sound mostly due to difficult respiration by narrowed airway passages
- *Runny nose:* nasal discharge of any mucus-like material coming out of the nose (Rhinorrhea)
- *Vomiting:* forcing the contents of the stomach up through the esophagus and out of the mouth
- *Diarrhoea:* at least three loose-to-watery bowel movements in a 24 h period that exceeded the child’s usual daily stool frequency by two or more stools and perceived as diarrhoea by the mother (WHO definition: “passage of loose or watery stools at least three times in a 24 h period“; emphasis on importance of change in stool consistency rather than frequency; usefulness of parental insight in deciding whether children have diarrhoea or not; see WHO: The treatment of diarrhoea: a manual for physicians and other senior health workers, WHO/CDR/95-3. Geneva: World Health Organisation, 1995)

Severity of illness episodes is explored by means of parameters such as

- Duration of illness symptoms (number of days; illness period is considered as finished if the child is again without symptoms and shows a normal activity level; after recovery, another illness is considered as new period if the symptoms occur at least three days after the stated time of recovery from a previous episode).
- Medication required as therapy (paracetamol, antibiotics, others) and their duration of use (number of days)
- Number of visits to hospital/practitioner
- Information if a physician has been visited who confirmed a diagnosis of infection
- Absence from day-care due to illness (number of days)
- Necessity of hospitalisation and respective duration (number of days)

Occurrence of allergic symptoms with focus on the skin (atopic dermatitis) will be checked during the clinical examinations:

- Criteria for diagnosis: typical morphology and distribution, pruritus (sign of scratching), chronicity (duration at least 14 d and/or chronically relapsing)
- Severity to be scored according to SCORAD

The skin should also be checked during this examination for eczema and urticaria.

Further secondary parameters are those gained through analyses of biological samples, i.e. stools and blood. As biochemical blood/plasma parameters of immune status, at least in a subgroup of infants, including vaccination response, following parameters of humoral or cellular status or mediators will be checked:

- Plasma antibodies: IgG, IgE, specific antibody titres (as response to vaccination; as allergy markers: antibodies against cow's milk, cat, soy, wheat and dust mite allergens)
- Lymphocyte subpopulations
- Cytokines (after ex vivo stimulation)

As stool parameters of gut health, immune status and inflammation, at least in a subgroup of infants, the following parameters will be analysed:

- CFU/g stools of Bifidus sp., Lactobacilli sp., and pathogenic micro-organisms
- faecal pH
- faecal sIgA

6.3 Safety parameters

As anthropometric parameters will be measured:

- Body weight and crown-heel body length
- Occipito-frontal head circumference
- Mid-upper arm circumference
- Skinfolds (triceps and subscapular)

Parameters of acceptance, tolerance and gastrointestinal discomfort are

- Number of feedings / intake
- Regurgitation/reflux: spitting up (mild, effortless), posseting (moderate), vomiting (severe, forceful)
- Stool frequency; stool consistency; stool colour
- Flatulence (excessive air/gas in the intestine passed through the rectum); cramps (unpleasant sensation caused by abdominal contraction); colics (intermittent attacks of abdominal pain when the baby screamed and drew up his/her legs but was well between episodes)
- Nappy rash (redness of the skin confined to the area covered by the nappy)

6.4 Possible confounding factors

6.4.1 Obstetric, medical and social data

- Maternal factors: age, parity, social class, height, smoking habit, marital stage, puerperal illness, previous medical illness
- Paternal and social factors: age, social class, number of rooms in the house, living conditions (urban / rural), smoking habit
- Infant factors: sex of child, date of birth (season!), birth weight, gestational age, mode of delivery (vaginally / caesarean section), Apgar score, neonatal jaundice, other illness before study entry, immunisation, number and age of siblings, illness in siblings, form of child care (home care, group care, day care centre)

6.4.2 Feeding pattern

- Full breastfeeding: breastfeeding for more than 4 months without any introduction of supplements (formula feeds, cow's milk, solid feeds; some juice or water will be not counted as such) before end of 4th month of life
- Partial breastfeeding: breastfeeding for more than 4 months with introduction of supplements before end of 4th month
- Early weaning: discontinuation of breastfeeding before end of 4th month
- Formula feeding: first formula feeding before study entry (full formula feeding = bottle feeding from birth onwards = no breastfeeding at all); after study entry all formula given is study formula (IF/FOF); after the second month of life on full study formula feeding
- Mixed formula feeding: first formula feeding before study entry (but not on full formula feeding = partial breastfeeding); after study entry all formula given is study formula (IF/FOF); after the second month of life still partial breastfeeding

7. STUDY DESIGN

The study will be performed as double-blind, placebo-controlled, randomised, prospective nutritional intervention study with a parallel group design.

8. STUDY DURATION

- Per subject: Infants will be recruited until the infant's age of 8 weeks at the latest and will be followed until the age of 1 year.
- Recruitment phase: Subject inclusion in the five participating centres should be finalised within about one year.
- Total study duration: The total study will last for about 2 years in each center. It will start in June 2005 (first center) – August 2005 (last center) and the clinical part (last infant finished the study) will be completed presumably in August 2007 (last center).

9. STUDY GROUPS

There will be two formula groups in the study:

- Group 1: Active Formula Group on full formula feeding after 2 months at the latest receiving a non-hydrolysed cow's milk based formula containing an immunomodulating mixture of neutral and acidic oligosaccharides. To avoid any immunological influence by other ingredients, the formula will not contain any other special supplementation such as probiotics, LCPUFA, or nucleotides.
- Group 2: Control Group on full formula feeding after 2 months at the latest receiving as placebo a standard non-hydrolysed cow's milk based formula with the same composition as the active formula but without supplementation of oligosaccharides. Other ingredients with suspected immunological influence will also not be supplemented, i.e. the only difference between the two formulas is the presence or absence of the supplemented mixture of neutral and acidic oligosaccharides.

The allocation to the two formula groups will be performed in a randomised way according to the stratified randomisation table (see annex) at study entry.

As non-randomised reference group, infants on full breastfeeding for at least 4 months will serve.

10. STUDY SUBJECTS

10.1 Enrolment of subjects

Mothers and their babies in the first 8 weeks of life from the region of Berlin, Groningen, Milan, Naples and Zurich will be informed about the study and asked to make up their mind if they would like to participate. E.g., a good time to approach the mothers is a routine visit to their hospital/practitioner for a routine investigation of their infants at the age of 4-6 weeks.

Those healthy term born infants fulfilling all requirements of inclusion but not exclusion criteria (see para 10.2 and 10.3) and whose parents after having been informed about the study and after having thought about it for at least some days have given their written consent for participation of their child in the study shall be enrolled. Each mother-infant couple will get a sequential number at study entry and keep that number throughout the study.

Study infants will be continuously recruited until a total number of about 500 formula infants per group will be reached (see para 10.4).

To comply with ethical standards not to influence breastfeeding mothers in any way to use an infant formula instead, mothers will only be asked to be recruited to a study formula group if they cannot/do not fully breastfeed their babies and had at least fed one bottle of formula before (mothers on full breastfeeding may only enter the reference group). Alternatively, mothers with healthy term newborns may be informed about the study (e.g. by a respective flyer containing also a contact telephone number), so that they actively can call back in case they would later on like to participate. Even after recruitment, mothers will not be influenced in any way in her decision to continue with breastfeeding as long as possible and therefore will also be followed within the study if she is able to breastfeed (at least partially) after their infants' age of 2 months (mixed-feeding group). Without ambivalence mothers will be told that breastfeeding offers clear advantages also by reducing the incidence of infections. She will not be told that infants will only be evaluated as formula infants in the per-protocol analysis if breastfeeding is finalized until the end of the second month of life.

By this procedure it should also be possible to exclude that the initial short breastfeeding period could be responsible for the outcome effect observed in the study. This assumption is supported by the results of the Dundee Breastfeeding Study which did not provide any evidence to suggest that brief periods of breastfeeding are sufficient to offer significant protection (Howie PW et al: Protective effect of breast feeding against infection. Br Med J 1990; 300:11-16). Nevertheless data on the duration and percentage of breastfeeding before and after study entry will be collected in order to test an influence as possible confounder also in the formula groups.

10.2 Inclusion criteria

- Healthy, term born infants (GA 37-42 weeks)
- with a normal birth weight (girls: 2.7-5.0 kg, boys: 2.9-5.2 kg),
- not older than 8 weeks of age when entering the study,
- not at high risk to develop an atopic disease (at least one parent or sibling with manifest atopic symptoms of hay fever, asthma or atopic dermatitis) which would require the feeding of an HA formula with hydrolysed protein according to current recommendations,
- and with no metabolic disorders requiring a special diet other than standard non-hydrolyzed IF/FOF.

10.3 Exclusion criteria

- Mothers suffering from hepatitis B, HIV or GBS (Guillain-Barré syndrome)
- Mothers taking antibiotics during breastfeeding
- Infants with known congenital diseases or malformations which would interfere with the study, e.g. gastrointestinal malformations, congenital or acquired immunodeficiency, etc.
- Study pre-feedings of the infants which could interfere with the study, e.g. non-cow's milk based formulas, HA formulas, probiotic formulas, etc.
- Infants with actual or previous illnesses which could interfere with the study (see CRF) Screening).

10.4 Randomisation

Randomization to one of the two formula groups will be performed directly after study entry of those mothers who have fed their infants at least one bottle of infant formula. Randomization will be performed stratified according to center. I.e., for each center a separate randomization list will be provided. All other potential confounders, such as obstetric, medical or social characteristics, will be checked and adjusted for in the final data analysis.

Time-balanced randomization will be performed with the software RANCODE (IDV, Gauting; seed numbers randomised by reaction time) with a random permuted block size of 4 so that after 4 infants each the same number of infants per formula group is reached. By this procedure the time influence (season etc.) is evenly distributed between the randomised group. An example of a randomization table is given in the annex.

10.5 Number of subjects and study power

Starting with 500 formula infants per group, it is assumed that about 35% will be lost for the per-protocol-analysis due to continuation of breastfeeding beyond the second month of life and further about 15% due to different dropout / withdrawal reasons so that 250 infants per formula group fully completing the study should be achieved.

With those 250 formula infants per group completing the study without violation of the protocol, a reduction of the mean number of febrile episodes by 1 which is considered as clinically relevant reduction (from 4.5 to 3.5, SD 3.5 episodes), compared to placebo can be proven with 93% power in an one-sided unpaired t-test or 89% in a two-sided unpaired t-test.

Statsol nQuery Advisor 5.0: Two group t-test of equal means (equal n's)

nQuery Advisor - [MTT0-1]

Two group t-test of equal means (equal n's)

	1	2	3	4	5	6	7	8
Test significance level, α	0,050	0,050	0,050	0,050	0,050	0,050	0,050	0,050
1 or 2 sided test?	1	2	1	2	1	2	1	2
Group 1 mean, μ_1	4,500	4,500	4,500	4,500	4,500	4,500	4,500	4,500
Group 2 mean, μ_2	3,500	3,500	3,500	3,500	4,000	4,000	4,000	4,000
Difference in means, $\mu_1 - \mu_2$	1,000	1,000	1,000	1,000	0,500	0,500	0,500	0,500
Common standard deviation, σ	3,000	3,000	3,500	3,500	1,500	1,500	2,000	1,500
Effect size, $\delta = \lvert\mu_1 - \mu_2\rvert / \sigma$	0,333	0,333	0,286	0,286	0,333	0,333	0,250	0,333
Power (%)	98	96	93	89	98	96	87	96
n per group	250	250	250	250	250	250	250	250

For Help, press F1 | 1,500000 | AUTO RECALC OFF

The assumption of at least 4.5 infection episodes is based on a study investigating the frequency, nature and severity of infections throughout the first 3 years of life in relation to three types of child care arrangement (home care, group care (2-6 children), and day care (> 6 children)). In this study it could be observed that the infection rates ranged from 4.5 episodes in the first year of life for infants with home care to more than 7 episodes for infants in day care centers (Wald ER et al. 1991. J Pediatr 118:509-514). Among the different illnesses, upper respiratory tract infection (URTI) was most prominent with 3.8 episodes for infants with home care up to 6.3 episodes for infants in day care centers in the first year of life.

The assumption of a standard deviation of maximum 3.5 episodes is based on the same publication in which the above described results were observed with standard deviations between 2 (home care) and 3 episodes (group care).

10.6 Compensation to study subjects

Mothers randomised into a formula feeding group will receive the respective study formula free of charge throughout the study, i.e. until their infants are 1 year of age.

Mothers participating in the reference group with full breastfeeding for at least 4 months will receive a Numico local follow-on formula for the second half of the first year free of charge –if needed and requested.

At the end of the study and after the planned blood drawings in the subgroups, the respective infants (including breastfed infants in the reference group) will receive a small present (e.g. a cuddle toy).

Parents will not get any financial compensation for their participation in the study.

11. STUDY TEST PRODUCTS

11.1 Basic product composition and packaging / blinding

Group 1 (Active Formula Group): Non-hydrolysed cow's milk based formula containing an immunomodulating mixture of

- Neutral oligosaccharides: galacto-oligosaccharides and fructo-oligosaccharides (inulin) in a ratio of 9:1 and a dosage of 0.68 gram per 100 ml
- Acidic oligosaccharides: pectin hydrolysate, dosage of 0.12 gram per 100 ml

Group 2 (Control / Placebo Group): Standard non-hydrolysed cow's milk based formula with the same composition as the active formula but without supplementation.

To avoid any immunological effect by other ingredients, both formulas will not contain other supplementations with suspected immunological influence such as probiotics, LCPUFA, or nucleotides. The only difference between the two formulas is the presence or absence of the supplemented mixture of neutral and acidic oligosaccharides.

Formulas will be supplied as powder in 400 g tins. The infants will receive starting formula (IF) until the age of 6 months and follow-on formula (FOF) thereafter. The composition of the products is given in the appendix.

The labels on the tins will be multi-language labels (English, Dutch, German, Italian) to cover all languages used in this multi-centric study. Examples are given in the annex. As the labels are anonymous neither the clinicians / nurses / mothers who give the feedings to the infants nor the persons performing the examinations and the statistical evaluations of the data will know about the identity of the formulas.

The allocation of the two blinded formula tin variants, only differing in a small hidden detail, to the individual infants will be performed by persons not involved in the study (hospital pharmacy). According to the randomisation list provided by NUMICO, the formula tins will be allocated to the respective subject and labelled with the respective randomisaton number on the lid (numbered labels provided by NUMICO). For unblinding of study subjects –if necessary*- a sealed envelope is provided by NUMICO with a letter (example see annex) containing the allocation code (information how by means of the hidden detail the formula can be identified).

*The unblinding of a study subject should be left to the case that an unexpected serious adverse event occurs which requires the knowledge about the feeding regimen as basis for any decision on the therapy.

11.2 Preparation, administration and dosage

The method of preparation is similar to that of other infant formulas: addition of one level scoop of powder to the defined amount of water (boiled, lukewarm). For the feeding regimen, the official guidelines of the Ministry of Health are used. The frequency of formula feeding and the amount per feeding shall be ad libitum.

NUMICO as manufacturer of the products used during the study confirms that every quality control measure has been taken to assure correct composition and that the products have passed security clearance without objections. Expiry dates are im-printed on the packages / labels. However, normal shelf life can only be guaranteed if the product is appropriately stored:

Temperature: room temperature; avoid local heat (exposure to sun, radiators, etc.)
- Humidity: Close pack carefully after every use; do not store in rooms commonly known for higher humidity (kitchen, laboratories, etc.)
- Safety: to avoid misuse of trial material by inexperienced personal not involved in the study keep material at a respective safe place, if possible under lock and key.

The study starting formulas are indicated for feeding of full-term infants from birth onwards in cases where breast feeding is not possible, or the supply of breast milk is insufficient. For continuation of formula feeding beyond the infants' age of 6 months, follow-on formulas (FOF) are indicated.

No particular contraindications are defined. Contraindications, however, exists as for all cow's milk based infants formulas (cow's milk protein allergy, lactose intolerance, galactosemia, glucose-galactose-malabsorption).

11.3 Assessment of feeding compliance

Compliance will be evaluated by using the food intake information as registered in the questionnaires.

In infants providing stool samples the intake of active formula can be checked by stool analysis, e.g. excretion of non-digested oligosaccharides.

11.4 Logistics of product supply

NUMICO will supply the investigators with formulas on demand. At the study centres, the study formulas will be kept by the investigators, who agreed to store the formulas. A dispensary record of the study formulas issued and of any material returned will be kept. Unused formulas will be returned to Numico Research.

Study formula for 4-8 weeks will be given to the mothers in advance in case they started formula-feeding (partially or completely). Once they have started, sufficient supply of formulas is guaranteed by NUMICO for the first year of their infant's life.

11.5 Additional feeding recommendations

Mothers are encouraged to breastfeed their infants for at least 4 months, preferably 6 months. The only substitute or supplement in case of insufficient breastfeeding is the randomised study formula. Parents are asked not to introduce weaning foods until their infants have reached the age of 4 months.

12. STUDY PROCEEDING IN DETAIL

12.1 Enrolment

Parents and their infants are potentially eligible if they seek medical attention with one of the participating hospitals/practitioners, e.g. for routine investigation at their infant's age up to 6-8 weeks. They will receive a detailed oral explanation about the purpose of the study and will be supplemented with an explanatory letter. Alternatively, mothers with healthy term newborn babies may be informed about the study (e.g. by a respective flyer containing also a contact telephone number), so that they actively can call back in case they would later on like to participate. If the parents or guardians have had enough time (at least some days) to think over to participate, infants up to the age of 8 weeks will be checked in a screening process for inclusion and exclusion criteria. If they fulfill the defined inclusion but not exclusion criteria and if the parents have signed the written informed consent form, they will be recruited.

12.2 Study groups

If an infant has already been fed with at least one bottle of an accepted standard infant formula (cow's milk based formula with non-hydrolysed protein, no probiotics included), this infant will be recruited to the formula groups. Randomization between the two formula groups will take place immediately according to the prepared center-specific randomisation schedule (example see annex). If the infant is on full breast-feeding at study entry, it will be allocated to the non-randomised reference group. The latest possible inclusion day is at 8 weeks of age.

12.3 Per-protocol- and intention-to-treat-analysis

The protocol foresees for the formula infants that they reach full formula feeding (FF) after 2 months of age at the latest and for the reference infants that they should stay on full breastfeeding (BF) for at least 4 months. Formula infants should cover all formula feedings during the study duration, i.e. till the infant's age of one year, with study IF/FOF. Deviations from this protocol will result in exclusion from the defined formula and breastfed groups with respect to the intended per-protocol-analysis (PP) after the study's end. Formula infants receiving breast milk beyond the age of two months will be evaluated in the per-protocol analysis as members of a mixed-feeding group (duration and estimated amount of breast milk intake will be recorded and statistically checked). For the intention-to-treat analysis (ITT), all infants having been randomised will be evaluated according to the initial group allocation.

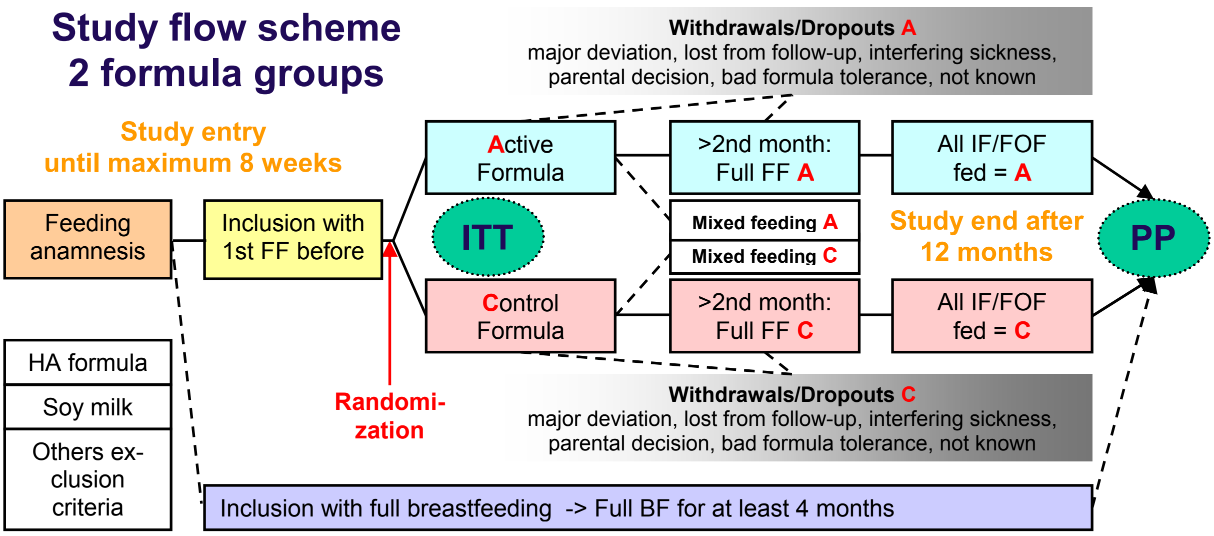

12.4 Examination day 1

The most important examinations will be performed at 5 examination days throughout the study. Day of study entry is defined as *examination day 1*. Questionnaires will be filled in with data on the mother incl. age, demography and socio-economic status, occupation, general health, nutritional status, delivery etc. Data on the feeding pattern will be collected as well (basal questionnaire). The clinical examination of the infant will include anthropometry but to check for pre-existing illnesses which might interfere with the study also the assessment of atopic dermatitis and other skin symptoms (eczema, urticaria, others) as well as the assessment of respiratory (rhinitis, wheezing, etc.), GI tract (vomiting, colics, diarrhoea) and general symptoms (failure to thrive, irritability, food refusal).

12.5 Additional examinations in study subgroups

If the mother agreed that her child may provide stool samples for check of stool parameters on gut health and immune status throughout the study (examination day 2-5), faeces should be collected for later quantitative and qualitative bacterial analysis, pH measurements as well as analysis of faecal sIgA. Thus she will receive stool containers provided by NUMICO in which she should collect some fresh stool sample from her infant’s first nappy of one day in the week before the next examination day (to be kept frozen and to be delivered by the mothers at next examination day). If the mother agreed that her child may also provide two times a small blood sample (2-3 ml) for more direct check of the immune status, a blood sample should be drawn for later analysis of biochemical blood/plasma parameters at the infant’s age of 6 and 12 months. If infants are seriously ill or feverish at the time of the blood sampling or if they used antibiotics in the 2 weeks preceding the blood sampling (as indicated by the parents or concluded from the anamnesis), the sampling point can be postponed for up to 2 weeks.

12.6 Interviews

During the study period every 2-4 weeks parents are interviewed according to a structured protocol on the health status of their infants incl. feeding pattern, acceptance and tolerance, possible complaints etc. (follow-on questionnaire). To help accuracy of recall, mothers will be supplied at study entry with a diary containing record cards for feeding data (number of breast feeds, number and types (volume intake) of bottle feeds, number of juice and water feeds, number of solid feeds, dates of first bottle feed, last breast feed, first solid feed) as well as any symptom of their infants' illness and respective medication (see diary CRFs in the annex). The close contact to the mother (two-weekly in the study beginning up the infant's age of 4 months and four-weekly afterwards) will be guaranteed by a mix of personal contacts (visits in the clinic or at home) and intermittent telephone calls. At the end on each individual's study period all information gathered by home visits and telephone interviews should be retrospectively scrutinized by comparisons with the general practice records as this has been identified to minimise detection bias (Howie PW et al: Protective effect of breast feeding against infection. Br Med J 1990; 300:11-16). Whenever there is any doubt whether a disease episode fulfils the agreed definitions or not, a decision is to be made on the basis of information available by the local responsible clinician.

12.7 Further examination days

Repetition of the examinations and further sample drawings are planned for the infant's age of about 2 months (*examination day 2*, stools), about 4 months (*examination day 3*, stools), about 6 months (*examination day 4*, stools) and 12 months, i.e. at study end (*examination day 5*, stools and blood).

12.8 Vaccination / vaccination response

A uniform vaccination routine using HEXAVAC (to be provided by NUMICO) will be possible in the two Italian centers with vaccinations after 3, 5, and 11 months. The German center will also use HEXAVAC, however with another timing (2, 3, 4, 11 months, see study schedule overview below). The vaccination routine in The Netherland and Switzerland differ from that according to country specific regulations regarding vaccination against Hepatitis B. The measurements of the immune status at examination day 5 (12 months) will include the measurement of the antibody response to the specific type of vaccination in the subgroup of HEXAVAC vaccinated infants having provided a blood sample.

12.9 Special recheck of feeding group

At the infant's age of 8 weeks the feeding regimen of infants in the formula groups are rechecked with special importance. Only those formula infants who reached full formula feeding will be evaluated in the per-protocol analysis as formula infants, those who are still on partial breastfeeding as mixed formula infants. Similarly, infants recruited on full breastfeeding will be rechecked with special importance at 4 months if they are still on full breastfeeding to be evaluated later on as fully breastfed infants. Final recheck of adherence to feeding regimen is at study end.

12.10 General assessment of each study subject during and after the study

The principle investigator should give -based on his clinical experience and knowledge about the respective study parameters of each study subject- his personal opinion on the anthropometrical development of the infant as well as the acceptance and tolerance of the respective study formula by filling in a general assessment form (see annex) after 4, 6 and 12 months of each study subject's age. If an infant finishes the observation period before the regular end of the individual study period the form has also to be filled in as final assessment.

12.11 Provision of formulas

At study entry mothers and their infants in the formula groups receive adequate amounts of respective infant formula powder to cover the first study period, i.e. for the next 2-4 weeks. Afterwards, the packages needed to cover the next study period of 4-8 weeks will be delivered to the participating families by the health visitors on the occasion of planned home visits or will be given to the mothers when coming to planned examinations of the study.

12.12 Study schedule overview

The planned schedule is depicted in the following table.

Age of infant (w) [1]	0 - <8	8	10	12	14	16	18	20	22	24	26	28
Personal contact (clinic / home visit)	X (U2/3)	X				X (U4)				X (U5)		
Interview (home visit or telephone contact)			X	X	X			X				X
Examination day	1	2				3				4		
Informed consent	X											
Randomization [2]	X											
Clinical examin.	X	X				X				X		
Basal questionnaire	X											
Follow-on quest.		X	X	X	X	X		X		X		X
General assessment by the PI						X				X		
Special recheck of feeding group		X (FF)				X (BF)						
Vaccination with HEXAVAC [4]		D		I; D		D		I				
Stool sample [3]		X				X				X		
Blood sample [3]										X		
Formula provided [2]	IF	IF		IF		IF		IF		FOF		(FOF)

Age of infant (w) [1]	30	32	34	36	38	40	42	44	46	48	50	52
Personal contact (clinic / home visit)												X
Interview (home visit or telephone contact)		X		X		X		X		X		
Examination day												5
Clinical examin.												X
Follow-on quest.		X		X		X		X		X		X
General assessment by the PI												X
Special recheck of feeding group												
Vaccination with HEXAVAC [4]								I; D				
Stool sample [3]												X
Blood sample [3]												X
Formula provided [2]		FOF		(FOF)		FOF		(FOF)		FOF		

[1] time window of 1 week e.g. 12 weeks = 12th weeks; 16 weeks = 16th weeks [2] formula groups only
[3] subgroups (specific written informed consent) [4] NL & CH according to country specific regulations
IF infant formula, FOF follow on formula, FF formula feeding, BF breastfeeding

13. DATA AND SAMPLE COLLECTION AND LABORATORY ANALYSES

13.1 Number of febrile episodes (primary outcome parameter)

The primary outcome parameter, i.e. the number of febrile episodes (peak rectal temperature at least 38.5 °C) in healthy term born infants during the first year of life will be assessed by structured interviews recalling data filled in by the mother in a diary with record cards and afterwards checked against the general practice records.

13.2 Symptoms of illness (secondary outcome parameter)

Symptoms of illness (cough, wheeze, runny nose, vomiting, diarrhoea, others) indicating a viral or bacterial infection are recorded by the mothers by filling in prepared diaries and are recalled by interviews during the 2-4 weekly personal or telephone contacts. Information on duration of symptoms and related medication, resulting absence from day care or even hospitalisation etc. will be collected as well.

13.3 Faecal parameters (secondary outcome parameter)

For each examination day, a stool sample of approximately 2-5 g faeces will be collected into stool containers provided by NUMICO Research and stored at -20°C for later analysis. The microbial composition of the faecal samples will be tested in those samples in selected subgroups directly by fluorescent in situ hybridisation (FISH) using specific 16S rRNA-targetted oligonucleotide probes for e.g. *Bifidobacteria, Lactobacilli, Bacteroides, E. coli* and *Clostridia.* Amounts are expressed as percentage of the total number of bacteria. FISH analyses will be organised by NUMICO Research.

As further faecal parameters of gut health and immune status, faecal pH will be measured and faecal antibodies (sIgA) determined with standard laboratory techniques (Elisa, FACS, RT-PCR).

13.4 Plasma parameters (secondary outcome parameter)

At examination days 4 and 5, i.e. at the infants' age of 6 and 12 months, 2-3 ml venous blood will be obtained for analysis of immune status from those infants whose parents agreed to participate in study subgroups providing blood samples of the infants during the study. Immediately after blood sampling, the blood cells will be separated, prepared and stored at -20°C stored for later analysis of different blood cells and subpopulations The remaining plasma will be stored frozen till later analysis of plasma antibodies by ELISA technique. All analyses will be organized by NUMICO in collaboration with the centers.

Following parameters will be investigated:

- Plasma antibodies: IgG, IgE, specific antibodies (response to vaccination / allergy markers: antibodies against cow's milk, cat, soy, wheat, dust mite allergens)
- Lymphocyte subpopulations
- Cytokines (after ex vivo stimulation)

As most of the study parameters will be influenced by infectious disease and/or antibiotic treatment, stool and blood samples should be only obtained after a period of two weeks free of infectious symptoms and/or antibiotic treatment. To fulfil this requirement, it is allowed to postpone the sampling for a maximum of two weeks.

13.5 Allergy symptoms (secondary outcome parameter)

The clinical examinations of the infants will include also the assessment of allergic symptoms with focus on the skin.

Occurrence and severity of atopic dermatitis will be assessed using the SCORAD index (The European Task Force on Atopic Dermatitis. Consensus Report of the European Task Force on Atopic Dermatitis: severity scoring of atopic dermatitis, the SCORAD index. Dermatology 1993; 186:23-31; Oranje AP, Stalder JF, Taieb A, Tasset C, De Longueville M. Scoring of atopic dermatitis by SCORAD using training atlas by investigators from different disciplines. Pediatr Allergy Immunol 1997; 8:28-34).

The skin will also be checked during this examination for eczema and urticaria as well as other symptoms.

13.6 Anthropometrics and other safety parameter

Growth parameters (for birth retrospectively collected) will be measured as part of the clinical assessment at study entry and at the infants' age of 2, 4, 6 and 12 months (study end) with standardized techniques: The naked infants are weighted on an electronic scale accurate to 1 g. Recumbent length is measured on a Harpenden stadiometer to the nearest 1 mm (one examiner holds the child's head and applies gentle pressure to bring the top of the head into contact with the fixed headboard; the second examiner extends the child's legs and, applying gentle traction, brings the movable footboard to rest firmly against the child's heels with the toes pointing directly upward). The occipital-frontal head circumference is measured using a paper or plastic tape measure or a flexible narrow steel tape to the nearest 1 mm (the tape is applied firmly around the head above the supraorbital ridges, covering the most prominent part of the frontal bulge anteriorly, and over the part of the occiput that gives maximum circumference). As indicator of body composition (regional fat and fat-free tissue), mid-upper arm circumference will be measured with a non-stretchable tape at the midpoint between the acromium and olecranon to the nearest 1 mm; triceps and subscapular skinfold thickness will be examined by means of a Holtain skinfold caliper calibrated to 0.2 mm. Only experienced personnel will perform the measurements. At any time, 3 measurements are taken for each growth parameter and the average value will then be considered. Actual chronological decimal age is used to calculate the standardized anthropometric indices (z scores). The z scores represent the distance in standard deviation (SD) units from reference growth values adjusted for age and gender derived from the Eurogrowth study (Haschke F, van't Hof MA, and the Euro-Growth Study Group. Euro-Growth References for length, weight, and body circumferences. J Pediatr Gastroenterol Nutr 2000, 31(suppl 1) S14-38).

Aside from the anthropometrical development of the infants, safety data on the incidence of gastrointestinal discomfort (flatulence / cramps / colics; occurrence of nappy rash) as well as on formula acceptance and tolerance (number of feedings / intake; spitting up / posseting / vomiting; stool frequency / stool consistency / stool colour) will be collected by the structured interviews throughout the study period.

Safety is also defined as absence of *unanticipated** adverse effects compared to baseline (pre-treatment) or “normal“ conditions as indicated by the control group. All *unanticipated* adverse events encountered during the study after treatment has started, whether considered related to treatment or not, will be reported on the subject’s record: intensity, relationship to test substance, action taken regarding test substance, and outcome to date (see Unanticipated Adverse Event Report Form the annex). Such preliminary reports will be followed by detailed descriptions when available.

*Unanticipated serious** adverse events thought to be associated with the study feeding will be reported to NUMICO Research Germany within 24 hours (Telefax 0049-6172-99-1862, Telephone Dr. G. Boehm 0049-6172-99-1320).

NUMICO Research has effected an insurance in connection with this study which meets the requirements as established, e.g., by the “De Nederlandse Wet Medisch Wetenschappelijk Onderzoek met Mensen”.

---

* *Unanticipated adverse events* are defined as any event, the specificity or severity of which is not consistent with those known from the risk information in the consent form (e.g. for blood samplings) or are reasonably expected to accompany the natural history and progression of the subjects’ condition throughout the study.
Serious adverse events are generally defined as events that result in any of the following outcomes: death, a life threatening experience; inpatient hospitalisation or prolongation of existing hospitalisation; a persistent or significant disability/incapacity; or a congenital anomaly/birth defect. In addition, events that may not result in death, be life-threatening, or require hospitalisation may be considered serious when, based upon appropriate medical judgement, they may jeopardise the subject and may require medical or surgical intervention to prevent one of the outcomes listed above.

14. STASTISTICAL ANALYSIS

14.1 Basic statistical procedure

Given the reasonably large sample size employing parametrical methods assuming a Gaussian distribution will be appropriate allowing for parametrical tests. If necessary, logarithmic transformations of skewed parameters are performed, e.g. for number of illness days.

The first step in the analysis will be a crude comparison between the two formula groups 1 and 2 testing the group effect (different formula) for

- quantitative variables by analysis of (co-)variance (ANOVA/ANCOVA)
- qualitative variables by Fisher exact test / Chi^2-test
- time-related variables (e.g. “time to infection“) by percental frequency curves according to Kaplan-Meier functions / Mantel-Cox log-rank test

One example is an analysis of variance with two groups (T-test) with the number of febrile episodes per child as the dependent variable and type of supplementation as a categorical independent variable. Another example is a Kaplan-Meier curve / Mantel-Cox log-rank test of the percentage of infants without infection throughout the study.

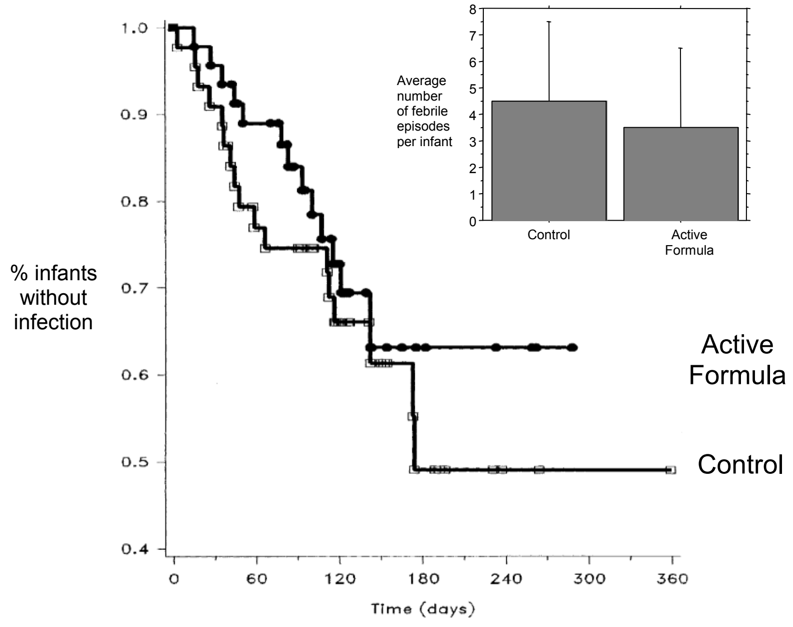

The second step is a series of univariate analyses to identify confounders by testing the possible influence of characteristics of the infants on the outcome parameters (to be expressed as odds ratio (OR) and 95% confidence intervals (CI)). Variables considered as potential confounders are, e.g., gender, birth weight, number of siblings, attendance to daycare, smoking in household, duration of breastfeeding, etc.

The third step is a multivariate analysis to identify interaction variables, i.e. confounders which interact with exposure (feeding active formula or placebo).

The final analysis is aiming at simultaneous control of possible confounders and interaction variables by multiple regression analysis with stepwise variable selection to achieve a model that is parsimonious (has only few variables) but maximizes the proportion of the explained variance across study groups. Stepwise procedure:

- baseline model including exposure and core variables,
- stepwise introduction of two-way interaction terms involving exposure (p value to enter 0.05),
- elimination of variables not involved in significant interactions in the multiple regression model (change in exposure odds ratio < 5%).

14.2 Descriptive analyses

For all *interval data* obtained at any date of measurement (e.g. quantitive data on faecal flora or anthropometric data) descriptive statistics will be computed for each feeding group including graphical analyses (scattergrams, single development curves, boxplots). If necessary, logarithmic transformation of data will be performed for homogenisation of variances (e.g. bacterial counts).

For *ordinal and nominal data* obtained at any date of measurement (qualitative data on faecal flora, acceptance and tolerance of feedings, single observations) percental frequencies will be calculated for each feeding group and visualised as bar and/or pie charts.

For time-related parameters (calculated data, e.g. time until occurrence of ... or time until ... was reached) graphical analyses will be performed for each feeding group by means of percental frequency curves according to Kaplan-Meier-functions.

14.3 Comparative analyses

If the data distribution of the respective parameter does not indicate a major deviation from Gaussian normal distribution, parametric tests will be used for group comparisons.

If the study hypothesis does not explicitly define the direction of group differences (e.g. parameter mean of group 1 significantly *less* (or *more)* ... than parameter mean of group 2) all tests will be performed two-sided.

Biometrical significance of group differences will be accepted if the null hypothesis (no group difference) is denied with an alpha-error of 0.05 (5%).

In case we assume equivalency for data, e.g. data on growth, acceptance, and tolerance in all groups), no correction for multiple comparisons by adjusting the level of alpha-error will be performed for those data in order to maintain the sensitivity of the tests to be used. However, for data assumed to be different between the groups, e.g. faecal flora, statistical analyses will be corrected for multiple comparisons (groups, time points) by adjusting the level of alpha-error according to accepted procedures, e.g. Bonfferoni.

14.3.1 Interval data

Inter-group comparisons will be performed for all interval data at any measurement point by using the analysis of variance (ANOVA) / T-test (if necessary to perform non-parametric tests -> Kruskal-Wallis test / Wilcoxon-Mann-Whitney U-test). In case of significance of the multiple group comparison, single group comparisons will be performed as post-hoc tests according to Fisher (if necessary to perform non-parametric tests -> Wilcoxon-Mann-Whitney-U-test).

For baseline adjustment, comparisons will be calculated not only using the raw data but also data differences (e.g. data at examination day 2 and 3 minus data at examination day 1) to account for individual variances.

Whenever needed, logarithmic transformation of data will be performed for homogenisation of variances prior to statistical analysis.

Within-group comparisons (e.g. examination day 3 versus day 1) will be conducted using the paired T-test (if necessary to perform non-parametric tests -> Wilcoxon-signed rank test).

14.3.2 Ordinal data

Ordinal data such as regurgitation and vomiting will also be analyzed by using the Kruskal-Wallis / Wilcoxon-Mann-Whitney-U-test. For within-group comparison the Wilcoxon-signed rank test will be used.

14.3.3 Nominal data

Qualitative data, e.g. tolerance data such as stool colour, will be analysed by using contingency table analyses of the coded raw data (Chi^2-test).

14.4 Correlations

Relationships between single interval data will be tested by regression analysis. If reasonable, logarithmic transformations of the data will be performed for linearisation of data relationships.

15. DOCUMENTATION

Case report forms (CRFs, see appendix) shall be used for recording all findings relevant for assessment of the selected parameters (parallel documentation of hospital's patient records).

For each form on which information is entered, the patient's initials, study number, date of birth, sex, and supplementation group shall be entered in the appropriate space. The CRFs shall be neatly handwritten, preferably with a black or blue ink ballpoint pen.

Errors must be corrected by drawing a single line through the incorrect entry and writing in the new value positioned as close to the original as possible. The correction must then be initialled and dated by the authorised individual making the change. In no case obliterate, write over, or erase the original entry when making a correction.

As soon as possible after the end of each infant's participation in the study, the CRFs shall be completed and signed by the principle investigator. Copies will be handed over to the local study manager for review of completeness. The investigator will maintain adequate records to enable the conduct of the study to be fully documented.

Protocols, subjects' records, originals of test result reports, formula dispensing logs, correspondence, records of informed consent and other documents pertaining to the conduct of the study must be kept on file by the investigator for a period of time specified by local law for the preservation of hospital subject documents. No study document should be destroyed without prior written agreement between NUMICO Research and the investigators. Should the investigators wish to assign the study records to another party, or move them to another location, NUMICO Research must be notified first.

The investigator shall supply NUMICO Research on request with any required background data from the study documentation or clinic records. This is particularly important when subjects' records are illegible or when errors in data transcription are suspected. In case of special problems and / or governmental queries, it is also necessary to have access to the complete study records, provided that subject confidentiality is protected.

Samples and questionnaires will be blinded in order to maintain confidentiality of trial documents and subject records and data will be maintained under secure conditions. For long term storage of any clinical material consent will be obtained. The investigators assure that subject's anonymity will be maintained. On all documents submitted to NUMICO Research, patients will not be identified by their names, but will be pseudonymized by an identification code, e.g. study number and initials. The investigator will keep a subject enrolment log showing codes, names and addresses. Documents not for submission to NUMICO Research, e.g. subject written consent forms, will be maintained by the investigator in strict confidence.

16. CLINICAL MONITORING

The principal investigator is in charge with guaranteeing continuous monitoring by supervising research nurses and physicians he/she has authorised. All findings recorded during clinical monitoring will be retained for at least 1 year and made available upon request of a professional NUMICO Research representative during a monitoring visit to obtain clinical background information for data evaluation.

17. CONFIDENCE AND PUBLICATION

Any information concerning the products and the test results of the study described above is treated as strictly confidential and it is not permitted to transmit or to publish them without written consent of NUMICO Research Germany. However, upon completion of study and integration of all results in the study report, the investigators may publish the results if the data warrant publication and if the number of study infants is sufficient for valid statistical analysis. Any decision in favour or against publication of study results is left entirely to the investigators. Nevertheless, in order to safeguard against disclosure of confidential information or misinterpretation of data, it is requested by NUMICO that any manuscript(s) for publication, texts of talks, abstracts or papers and similar material will be submitted to NUMICO for review, comment and approval prior to publication. In order to ensure that NUMICO will be able to make comments and suggestions where pertinent, material for public dissemination will be submitted to NUMICO for review thirty (30) days prior to submission for publication or review by a Programme Committee. In the unlikely event of any disagreement, e.g. to incorporate such comments and suggestions, the dispute shall be submitted to an independent third party (expert in this research field) mutually chosen by the parties. Such third party shall decide whether NUMICO's comments shall be included or not in the manuscript(s) and its decision shall be final and conclusive between the parties. NUMICO will reimburse any costs related to such third party assistance. The sequence of publications and the coverage of different aspects will be matter of discussion and common decision of the participating clinicians and NUMICO. It is understood, however, that no publications will occur until the publication of the main article for the study. The investigators may prepare the manuscript(s) with the assistance of NUMICO. Such assistance may include journal selection or writing or editing of the manuscript(s).

18. WITHDRAWALS AND DROPOUTS

Parents have the right to withdraw their infants from the study at any time for any reason. The investigator also has the right to withdraw subjects from the study for medical (or other sound) reasons. It is understood by all concerned that an excessive rate of withdrawals can render the study uninterpretable: therefore, unnecessary withdrawal of subjects should be avoided.

Refusal of specific study assessments or complete withdrawal from the study does not preclude a subject from subsequent evaluation. Unless they refuse, all subjects that have been randomised will be followed up.

If any subject fails to complete the study (withdrawal from the study, e.g. in case of revocation of parental consent, or dropout, e.g. in case of illness of study members*) the reason should be specified in writing by the supervising physician. If parents decide to withdraw their child from the study without giving any reason this should also be stated. If needed for clarification of dropout or withdrawal reasons telephone interviews are to be planned.

For the intention-to-treat analysis, results will be reported for all subjects as randomised. For the per-protocol analysis, however, withdrawals, dropouts, and non-compliers will be excluded. Furthermore, formula fed infants with partial breastfeeding beyond four study weeks will be evaluated as separate groups (mixed feeding).

In case of >30% missing data of an infant the concerned subject will be excluded from the per-protocol evaluation (dropout because of missing data). The same is true if the specific feeding regimen for an individual is discontinued before the study end (dropout because of violation of feeding regimen). For identification of non-completers as possible non-responders, however, the protocols (CRFs) of those cases should also be carefully checked. Incidental feeding of small amounts of food different from the prescribed feeding regimen will not lead to exclusion (incidental intake of infant tea, glucose electrolyte solution for some hours, or else).

* If patients fall ill during the study period, the main investigator will decide whether the disease requires withdrawal of the patient from the study. This will also depend on the type and dose of drug(s) provided for treatment.

19. PERSONAL RESPONSIBILITIES

19.1 Responsibility of the participating clinicians

The principal investigators ensure that this study is conducted in full conformance with the principles of the 'Declaration of Helsinki' and with the local laws and regulations, whichever affords the greater protection to the individual.

The principal investigators will obtain the local Ethical Review Committee or Institutional Review Board (IRB) approval before beginning of the study. This is necessary because this study proposal addresses sensitive ethical issues on several counts: involvement of infants and use of human tissues (blood and faeces) and use of personal data. The study documents must be sent to the Committee of Ethics of the participating universities for evaluation of protocol and any accompanying material provided to the subjects, such as information sheets or description of the study used to obtain informed consent. Approval from the committee will be obtained before starting the study, and will be documented in a letter to the investigator specifying the date on which the committee met and granted the approval. The principles of beneficence, non-malificence and the rights and autonomy of the study subjects will be observed at all times. Adequate written information will be provided to all subjects prior to their recruitment with the research fully explained and understood. Participants should be well informed before enrolment about the objectives, the procedures and protection of the privacy. Freely given informed written consent will be obtained prior to any clinical procedures.

Mothers will be capable of giving consent on behalf of their offspring. It will be clarified that consent is sought for research and that it is voluntary. The purpose of the research and procedures will be stated and the benefits, risks, discomforts and inconveniences.

It is the responsibility of the investigator to obtain written informed consent from the parents of subjects participating in this study, after adequate explanation of the aims, methods, objectives and potential hazards of the study. The investigator will also explain to the parents of subjects that they are completely free to refuse entrance into the study or to withdraw from the study at any time for any reason. Appropriate forms for documenting written informed consent will be provided by the investigator (see appendix).

The principle investigators will be responsible for conducting the study in accordance with the approved protocol and will ensure that the provided feeding will be administered only to subjects entering the study and under his personal supervision or under the supervision of the pre-designated investigators responsible to them, and will not be supplied to any other investigator or to any clinic for feeding to subjects.

Together with the local co-clinicians they will be responsible for preparing and maintaining adequate case histories designed to record observations and other data pertinent to the study. Copies of the signed CRFs will be forwarded to the local NUMICO Research monitor. If data are already fed into a computer, a copy of the data set should be handed over, too. After evaluation and common discussion of all study results the principle investigators will prepare a final report (alternatively one or more publications may be prepared).

All study related effects reported by the physician, dietician, nurse, or by the parents will be recorded and the local supervising physicians will pass details of all *unanticipated** untoward effects to NUMICO Research Germany. The nature of each unanticipated adverse experience, time, and relationship to the feeding should be established. Those of *serious* nature* thought to be associated with the study feeding will be reported to NUMICO Research Germany within 24 hours (NUMICO Research Germany, Dr. Günther Boehm, Telephone 0049-6172-99-1320, Telefax 0049-6172-99-1862). The study can be interrupted by the clinicians at any time if any unforeseen problems arise. However, NUMICO Research Germany will be informed within 24 hours about any intention to unblind a study subject or to interrupt/discontinue the study (if possible before final measures are taken).

19.2 Responsibility of NUMICO Research Germany

NUMICO will provide

- the appropriate agency clearance for the study (if necessary),
- the investigational products, appropriately labelled and packed,
- the randomisation schedule,
- the code envelope for unblinding of the trial material –if needed,
- the CRFs for recording all findings relevant for assessment of the selected parameters,
- financial remuneration for time and services as agreed upon according to the separate financial agreement.

All data transmitted to NUMICO Research Germany will be stored adequately.

---

* *Unanticipated adverse events* are defined as any event, the specificity or severity of which is not consistent with those known from the risk information in the consent form (e.g. for blood samplings) or are reasonably expected to accompany the natural history and progression of the subjects' condition throughout the study.
Serious adverse events are generally defined as events that result in any of the following outcomes: death, a life threatening experience; inpatient hospitalisation or prolongation of existing hospitalisation; a persistent or significant disability/incapacity; or a congenital anomaly/birth defect. In addition, events that may not result in death, be life-threatening, or require hospitalisation may be considered serious when, based upon appropriate medical judgement, they may jeopardise the subject and may require medical or surgical intervention to prevent one of the outcomes listed above.

19.3 Schedule of responsibilities

	NUMICO Research	Investigators	NUMICO country organisation
Protocol preparation	X	X	
Insurance	X		
Design Patient Information and Informed Consent Form	X	X	
Design CRFs, diaries	X	X	
IRB/MEC approval		X	
Microbiological analyses	X		
Analyses of blood	X	X	
Randomization	X		
Provision / distribution of product	X		X
Distribution of protocol	X		
Printing CRFs, questionnaires, etc	X		
Make scenario for the study		X	
Subject recruitment and conducting study		X	
Progress reports every 4 months		X	
Monitoring at an inclusion of 5, 20, 50, 100, 150 and 150 infants	X		X
Data entry	X	X	
Data analyses	X	X	
Final report	X	X	
Publication	X	X	

20. PROTOCOL CHANGES

Protocol modifications to ongoing studies which could potentially adversely affect the safety of participating subjects, or which alter the scope of the investigation, the scientific quality of the study, the experimental design, dosages, duration of therapy, assessment variables, the number of subjects treated or subject selection criteria, will be made only after consultation between the NUMICO Research co-ordinator and the investigators. Any proposed change has to be subject of a written protocol amendment.

21. APPENDIX

21.1 Composition of study formulas

The final details of the composition of the follow-on formulas are not yet definitive.

Nutrients per 100 ml	Unit	Experimental starting formula 13.6 g powder	Control starting formula 13.1 g powder	Experimental follow-on formula 14.5 g powder	Control follow-on formula 14.5 g powder
Energy	kJ	275	280	280	293
Energy	kcal	65	67	67	70
Protein	g	1.3	1.4	1.4	1.8
Fat	g	3.4	3.5	3.2	3.4
Linoleic acid	mg	520	397	489	489
α-Linolenic acid	mg	96	73	91	91
Total carbohydrates	g	7.3	7.5	8.2	8.1
Glucose	g	0.2	-	0.2	0.2
Lactose	g	7.0	7.4	7.9	7.9
Polysaccharides	g	-	-	0.1	0.1
Fibres	g	0.8	-	0.8	-
**GOS/FOS (9:1)**	g	0.68	-	0.68	-
**Acidic oligosaccharides**	g	0.12	-	0.12	-
Calcium	mg	52	52	78	88
Phosphorus	mg	26	26	48	48
Magnesium	mg	5	5	7	7
Sodium	mg	19	19	34	34
Potassium	mg	77	64	78	78
Chloride	mg	43	43	55	55
Iron	mg	0.5	0.5	0.71	1.26
Zink	mg	0.5	0.5	0.6	0.9
Copper	mcg	40	40	42	50
Manganese	mcg	7.3	7.3	11	11
Iodine	mcg	10	10	11	11
Selenium	mcg	2.5	1.5	2.5	1.5
Vitamin A	Mcg RE	51	60	55	55
Carotenoids	mcg RE	3.9	4.2	4.2	4.2
Vitamin D3	mcg	1.4	1.4	1.4	1.9
Vitamin E	mg α-TE	1.1	1.1	1.2	1.2
Vitamin K	mcg	4.9	5.1	4.9	4.9
Vitamin B1	mg	0.05	0.04	0.05	0.04
Vitamin B2	mg	0.11	0.11	0.11	0.11
Niacin	mg NE	0.76	0.76	0.75	0.75
Vitamin B6	mg	0.04	0.04	0.04	0.04
Vitamin B12	mcg	0.18	0.20	0.16	0.16
Vitamin C	mg	8.2	8.2	8.4	8.0
Biotin	mcg	1.4	1.7	1.8	1.8
Folic acid	mcg	11	11	11	11
Pantothenic acid	mg	0.33	0.30	0.34	0.30
L-carnitine	mg	1.0	-	-	-
Choline	mg	7.9	7.6	8.4	8.4
Taurine	mg	5.2	6.3	5.0	5.1

21.2 Multi-language labels of study formulas

21.2.1 Final version of the starting formula label

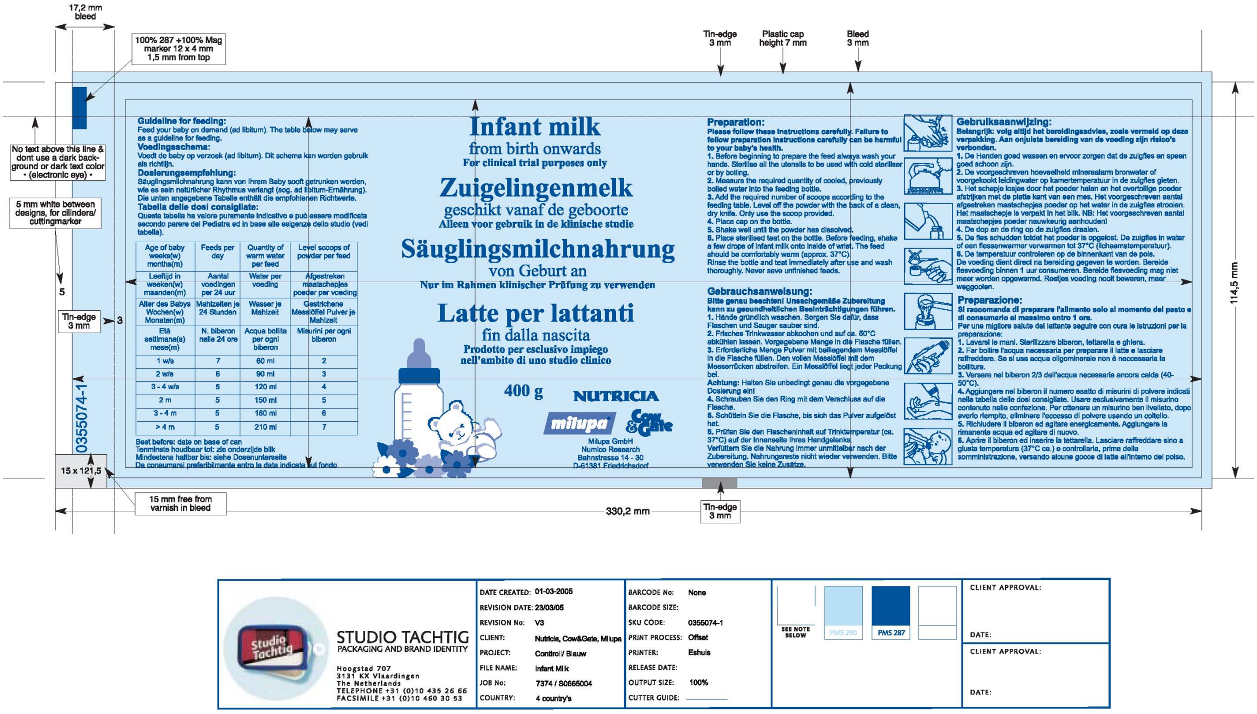

21.2.2 Final version of the follow-on formula label

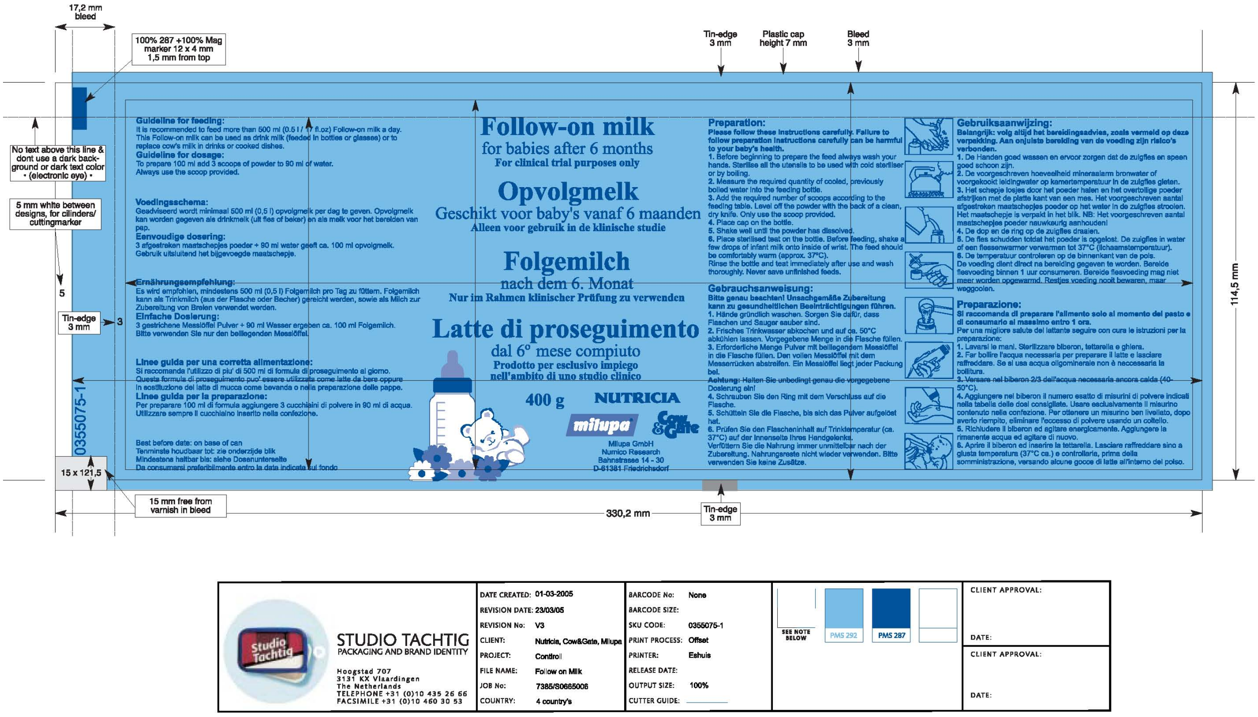

21.3 Guidelines on dosage and preparation of study formulas

The baby should be fed on demand, i.e. ad libitum.

The table for starting formulas below may serve as guideline for feeding from birth onwards.

Age of baby	Feeds per day	Quantity of warm water per feed	Level scoops of powder per feed
1 week	7	60 ml	2
2 weeks	6	90 ml	3
3-4 weeks	5	120 ml	4
2 month	5	150 ml	5
3 - 4 months	5	180 ml	6
> 4 months	5	210 ml	7

For the follow-on formulas, preparation is 3 scoops of powder + 90 ml water to get about 100 ml of feeding. It is recommended to feed more than 500 ml follow-on milk a day. The follow-on milk can be used as drink milk (feeded in bottles or glasses) or to replace cow's milk in drinks or cooked dishes.

On the following page the preparation of formulas is described and depicted by icons. These preparation instructions should be carefully followed to avoid any harm to the baby's health. After preparation of the feeding the formulas should be fed directly to the baby and should be consumed within one hour at the latest. Unfinished feeds must not be saved.

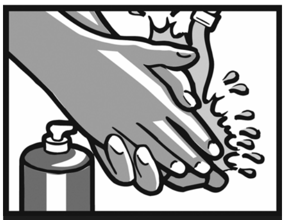

Before beginning to prepare the feed always wash your hands.

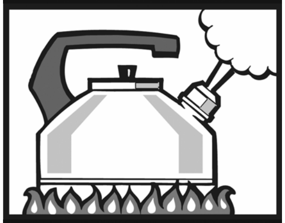

Sterilise all the utensils to be used with cold steriliser or by boiling.

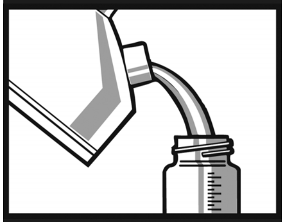

Measure the required quantity of the cooled previously boiled fresh drinking water into the feeding bottle.

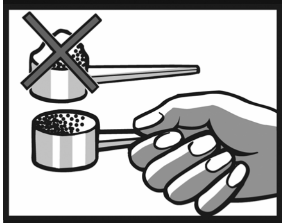

Add the required number of scoops according to the feeding table. Level off the powder with the back of a clean, dry knife.

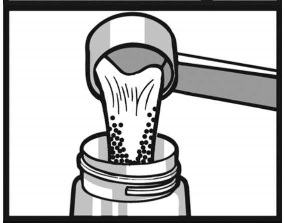

Only use the scoop provided.

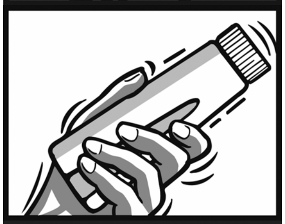

Place cap on the bottle and shake well until the powder has dissolved.

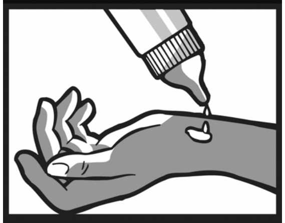

Place sterilised teat on the bottle. Before feeding, shake a few drops of infant milk onto inside of wrist. The feed should be comfortable warm (approx. 37°C).

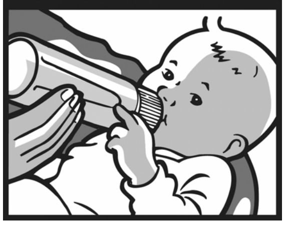

Please feed the formula directly after preparation. Rinse the bottle and teat immediately after use and wash thoroughly. Never save unfinished feeds.

21.4 Time-balanced randomization tables and recording of the enrolment dates

Randomization will be performed separately in each of the five centers.

Time-balanced randomization is performed with the software RANCODE (IDV, Gauting; seed numbers randomised by reaction time) with a random permuted block size of 4 so that after 4 infants each per stratum the same number of infants per formula group is reached. By this procedure the time influence (season etc.) is evenly distributed between the randomised group.

A randomization table like the example below will be used in each center, starting with the randomisation number 1. To allow small imbalances in the recruitment between the centers the randomisation numbers are established until a maximum of 500. The recruitment will stop in each centre after one year.

For infants on full breastfeeding which may be recruited to the reference group, the study numbers starting with 501 are reserved in each centre.

In order to avoid the occurrence of the same subject numbers in different centers which could probably result in mixing up of stool or blood samples during later central analysis, the subject numbers will be unique by reserving different ranges for each center. E.g., in Berlin the subject numbers will start with 1001 (1001 – 1500 = formula infants; > 1500 = breastfed infants), in Groningen with 2001, in Milan with 3001, in Naples with 4001, and in Zurich with 5001.

With respect to the intention-to-treat analysis of all study data in addition to the per-protocol analysis, no replacement of study numbers is intended.

Example: Randomisation of formula infants

No.	-> Group	Recr. Date	No.	-> Group	Recr. Date	No.	-> Group	Recr. Date	No.	-> Group	Recr. Date
1	A		51	A		101	B		151	B	
2	B		52	B		102	B		152	A	
3	B		53	B		103	A		153	A	
4	A		54	B		104	A		154	B	
5	A		55	A		105	B		155	B	
6	B		56	A		106	B		156	A	
7	A		57	B		107	A		157	B	
8	B		58	B		108	A		158	A	
9	B		59	A		109	B		159	A	
10	B		60	A		110	B		160	B	
11	A		61	A		111	A		161	A	
12	A		62	B		112	A		162	B	
13	A		63	A		113	A		163	B	
14	B		64	B		114	B		164	A	
15	B		65	A		115	B		165	A	
16	A		66	B		116	A		166	A	
17	B		67	B		117	A		167	B	
18	B		68	A		118	B		168	B	
19	A		69	B		119	A		169	B	
20	A		70	B		120	B		170	B	
21	B		71	A		121	A		171	A	
22	B		72	A		122	B		172	A	
23	A		73	B		123	B		173	B	
24	A		74	B		124	A		174	B	
25	A		75	A		125	B		175	A	
26	B		76	A		126	B		176	A	
27	A		77	A		127	A		177	A	
28	B		78	A		128	A		178	B	
29	B		79	B		129	A		179	A	
30	B		80	B		130	A		180	B	
31	A		81	A		131	B		181	B	
32	A		82	B		132	B		182	B	
33	A		83	B		133	B		183	A	
34	B		84	A		134	A		184	A	
35	B		85	B		135	B		185	B	
36	A		86	B		136	A		186	B	
37	A		87	A		137	B		187	A	
38	B		88	A		138	B		188	A	
39	A		89	A		139	A		189	A	
40	B		90	A		140	A		190	A	
41	B		91	B		141	A		191	B	
42	B		92	B		142	B		192	B	
43	A		93	A		143	A		193	A	
44	A		94	A		144	B		194	B	
45	A		95	B		145	B		195	A	
46	A		96	B		146	B		196	B	
47	B		97	B		147	A		…	…	
48	B		98	B		148	A		…	…	
49	A		99	A		149	A		…	…	
50	B		100	A		150	B		500	A	

21.5 Example of the sealed envelope for unblinding of a study subject

This letter has to be opened and read only in case of the necessity of unblinding the two blinded trial formulas in the clinical study testing the effect of formula feeding (IF and FOF) supplemented with a mixture of immunologically active neutral and acidic oligosaccharides on the incidence of febrile respiratory and gastrointestinal infections in healthy term born infants during the first year of life.

According to para 19.1 of the protocol (responsibility of the participating clinicians), the study can be interrupted at any time if unforeseen side-effects or results arise. In case of an unanticipated serious adverse event, if the knowledge about the feeding is needed for further therapeutic approach, the blinded formulas may be unblinded by opening this sealed envelope.

The different feedings may be identified by a small but specific detail on the tins:

If the xxx on the tins is XXX → the formula / follow-on formula is

the ACTIVE formula

If the yyy on the tins is YYY → the formula / follow-on formula is

the CONTROL formula

With this knowledge you are able to inform the principle investigator about the individual allocation of trial formula to the subject of interest. Please keep the general information strictly confidential.

According to the protocol, NUMICO Research Germany has to be informed within 24 hours about opening of the sealed envelope and the detailed reasons for it. Please phone Dr. G. Boehm (+49-6172-991320) or if you cannot reach him directly please contact Dr. Jelinek or Dr. Stahl. Overview contact addresses:

Numico Research Germany
Fax: ++49-6172-991862

Dr. Günther Boehm
Director Infant Nutrition Research
Phone: ++49-6172-991320, Email: guenther.boehm@milupa.de

Dr. Jürgen Jelinek
Head Data & Information Management
Phone: ++49-6172-991838, Email: juergen.jelinek@milupa.de

Dr. Bernd Stahl
Head Carbohydrate Research
Phone: ++49-6172-991496, Email: bernd.stahl@milupa.de

21.6 Letter to the parents for information about the study (local languages!)

You are being asked to participate in a clinical study.

Before you agree to take part in this study, the investigator for the study will tell you:

- Why the study is being done
- What will happen to you if you take part in the study
- How long you will be in the study
- Procedures that are going to be done only for this experiment
- The possible foreseeable risks, discomforts, and benefits of this research
- Alternatives to being in the study
- How your study records will be maintained and who will have access

If applicable to your taking part in this study, the investigator will tell you:

- Who you can call if you think you have been hurt by taking part in this study, if treatment for injury will be available, and who will pay for it
- The possibility of unforeseen risks
- If it will cost you anything to take part in this study
- Situations when the investigator may withdraw you from the study
- What happens if you decide to stop taking part in the study and who to call
- When you will be told about new findings which may affect your willingness to keep participating
- How many people will be in the study

In the following we like to summarize the most important points also in written form:

Newborn babies and young infants are especially vulnerable for infectious diseases. This is mainly due to their immature gastrointestinal tract and not yet fully developed immune system after birth. In breast fed infants the maturation of the gut and immune system is perfectly supported by the concerted action of active compounds of mother's milk. In fact, breastfeeding has a known protective effect, e.g. against gastrointestinal and respiratory infections as well as middle ear and urinary tract infections in early childhood.

One of these bioactive compounds in breast milk are the so-called oligosaccharides – soluble fibres which represent the third largest part of ingredients in human milk. As they are not or only very little absorbed in the gut, they serve as substrate for the beneficial flora in the colon. This effect is called prebiotic. Special oligosaccharides are suggested to have also further health-supporting influences, e.g. to be anti-infective and/or anti-allergenic and to positively modulate the immune system.

Based on recent research activities on human milk, a baby milk has been developed following partly the functional complexity of mother's milk by containing such oligosaccharides very similar to those in mothers' breast milk and in the same amounts. Thus it is thought that such a new baby milk may mimic the immune-mediated beneficial effect of breast milk on the healthiness of newborn infants. Apart from the special adaptation of the fibre composition, the new formula is identical to a standard baby milk that you would buy in the shop.

We wish to compare this new baby milk with normal baby milk. You should know that the new formula has already been successfully evaluated and that a normal growth development of the infants as well as a satisfying acceptance and tolerance of the feeding could be shown. The information to collect now will help us to confirm these findings in a much bigger cohort of infants all over Europe (about 1000 babies will be investigated within this study) and to show the suggested health benefits in terms of a reduced infection rate and other health parameters. As a reference group we also want to include breast fed infants in the study.

We would like to ask you to participate with your baby in this study. If you wish to bottle feed, you and your baby will be randomly assigned (much like the flip of a coin) to the new formula or a standard baby milk as control for the first year of life (you will have a 50% chance each of receiving the new or the control formula). As standard information we have to tell you that if you are assigned to receive the control formula, you will not receive the benefits of the new formula, if there are any, nor will you be exposed to its risks, if there would be any.

As far as you feed milk formula to your baby, please use the milk we will provide to you throughout the study. The milk will be supplied at grant in unmarked tins only to be used within this study. You and the physician and other persons doing the study will not know whether you are receiving the new or the control formula, so will you not know which milk your baby is getting but that information is available if it is needed.

Examinations of your baby for this comparison of the two baby milks will include measurements of growth (weight, height, head circumference etc.), allergy status (e.g. skin symptoms); respiratory symptoms like wheezing or cough as well as interviews concerning the feeding practice and acceptance and tolerance of the feeding (such as stool frequency and consistency).

These examinations will be continued in regular intervals until your baby's age of 12 months. This will be done also with the help of a nurse who will regularly visit you or call you to ask some questions in 2-4 weekly intervals. To let her have all information, we want to ask you to fill in a record to take notices for the coming interviews.

At 4 time points throughout the study we would like to ask you to provide a stool sample of your baby so that we can look for details of the stool flora.

As the development of the immune system can be well monitored by means of the response to the standardized vaccination of your baby, we also intend to investigate immune parameters in a small sample of blood (2-3 ml) at your baby's age of 6 and 12 months - if you agree. According to the standard information about the risk of venipuncture, we must indicate that the risks of drawing blood from a vein include discomfort at the site of puncture, possible bruising and swelling around the puncture site, rarely an infection, and, uncommonly, faintness from the procedure. However, by this special investigation the healthiness of your baby can be assessed in more detail. Nevertheless, the provision of a small blood sample is not a prerequisite for the participation in this study.

You may contact _(PI's name)___________ at (___) __________ at any time if you have questions about the research or if you think that you have been hurt by the research.

Although no special risks are foreseen by feeding your baby the newly developed or the control standard baby milk, clinical studies are covered by the insurance of the study sponsor. In this study Royal NUMICO N.V. has affected a clinical trial insurance via the Dutch company AON which covers property damages and health impairments to human subjects voluntarily participating in this clinical trial up to EUR 450,000 per human subject / EUR 5,000,000 (CHF for the Swisse insurance) for all claims combined in the aggregate of this trial*.

We would like to stress that if you agree to participate in this study we assure you that confidentiality of your personal data is strictly respected. The full name of you or your child is not released to anyone except the principal investigator and medical stuff members in charge (even in the publication(s) of the study results your identity will remain confidential). A direct access to the original medical records for verification of clinical trial procedure or data is only granted to the regulatory authorities, the IRB/IEC (Institutional Review Board/Independent Ethics Committee), the study monitor(s) and the auditor(s).

Signing the informed consent form means that the research study including the above information has been described to you and that you voluntarily agree to take part. If you agree to participate in this study, you will be given this written summary of the study and a copy of the signed informed consent form.

Should you not wish to take part in the study, then your baby will still receive the best care that we can provide. Should you decide to take part, you are still free to change your mind at any time and the baby can go back onto the milk of your choice. If you choose not to be in this study or if you quit being in the study at any time, there will be no penalty and no loss of any benefits you are entitled to.

---

* general exclusions from this clinical trial insurance are:
  - health impairments and/or further deterioration of pre-existing impairments that would have occurred or continued even if the subjects had not participated in the clinical trials are not covered by this insurance)
  - health impairments in connection with genetic engineering

21.7 Written consent of the parents for participation of their infant in the study (local languages!)

I state herewith that I have no objections against including my child _____________ born ________________ into the nutritional study (up to the infant's age of 1 year) under the guidance of Professor ___________________, aiming to evaluate a new infant formula containing prebiotic oligosaccharides in amounts found in human milk and thus closer to the composition of breast milk than conventional formulas.

I have been informed that the infants pertaining to the formula feeding groups of this study will be fed only standard infant formula containing a conventional or an improved carbohydrate blend. If I belong to the breast feeding group, I intend to exclusively breast feed for a time period of at least 4 months, without complementary feeding.

My child will remain under the care of Professor __________________ and will be examined at study entry and afterwards in regular intervals till the age of 1 year. Examination will include anthropometric measurements (e.g. weight, length, head circumference etc.) as well as interviews concerning the health status of my child and in addition the feeding practice, acceptance and tolerance of the feeding. During the study, I will undertake to fill in the diary as record for the regular interview to be conducted by a nurse of the hospital.

As I learnt that many of the suggested beneficial effects of the new formula might be due to the prebiotic effect on the gut microflora, I agree to the planned study procedure to collect some stool samples from my child.

I have been informed that the participation of my child in the study is voluntary and may be discontinued at any time.

I authorise my doctor to review my child's medical records if necessary for purposes of the study. I was assured that confidentiality of personal data is strictly respected and that the full name of my child is not released to anyone except the principal investigator and medical stuff members in charge. I agree that a direct access to the original medical records for verification of clinical trial procedure or data is granted to the regulatory authorities, the IRB/IEC (Institutional Review Board/Independent Ethics Committee), the study monitor(s) and the auditor(s).

_________ _______________ ___________________________

Date Place Signature of person giving consent

_________ _______________ ___________________________

Date Place Signature of person obtaining consent

I further agree to provide a small amount of venous blood (2-3 ml) of my child at the age of 6 months and 12 months (i.e. the end of the study period), to allow for investigation of the beneficial effect of the new formula on the immune system by means of biochemical immune parameters. I was informed about the possible risks of venipuncture, i.e. discomfort at the site of puncture, possible bruising and swelling around the puncture site, rarely an infection, and, uncommonly, faintness from the procedure.

Date	Place	Signature of person giving consent
Date	Place	Signature of person obtaining consent

21.8 Study insurance documents

All study insurance documents are available in NUMICO as signed originals. Scans of these originals are electronically available at each study sites and can be provided on demand:

- Master insurance certificate for Germany, Switzerland and Italy (English version)
- German version of the insurance certificate for Germany
- German version of the insurance certificate for Switzerland
- Italian version of the insurance certificate for Italy
- Dutch insurance certificate for The Netherlands (for the Netherlands a separate local insurance had to be organized for this study)

21.9 CRFs and diaries

Case report forms and diaries for the mothers have been elaborated, translated and printed and are therefore available in local languages in each center. CRFs and diaries cover chronologically the whole individual study duration of each study subject.

The English versions (master files) of the CRFs and diaries are electronically available.

For the classification of stool consistency by the mothers, 3 of the 5 stool consistency possibilities, i.e. the three middle categories, are provided as exemplified photographs in the diaries to assist the mothers during the 3 days' tolerance checks throughout the study.

Furthermore, several explanations and definitions are included in the diaries to support the mothers in filling in the questionnaires.
